# Dual-targeting of *Arabidopsis* DMP1 isoforms to the tonoplast and the plasma membrane

**DOI:** 10.1371/journal.pone.0174062

**Published:** 2017-04-06

**Authors:** Alexis Kasaras, Reinhard Kunze

**Affiliations:** Institute of Biology—Applied Genetics, Dahlem Centre of Plant Sciences (DCPS), Freie Universität Berlin, Berlin, Germany; Beijing Forestry University, CHINA

## Abstract

The reports of dual-targeted proteins in plants have steadily increased over the past years. The vast majority of these proteins are soluble proteins distributed between compartments of the non-secretory pathway, predominantly chloroplasts and mitochondria. In contrast, dual-targeted transmembrane proteins, especially of the secretory pathway, are rare and the mechanisms leading to their differential targeting remain largely unknown. Here, we report dual-targeting of the *Arabidopsis* DUF679 Membrane Protein 1 (DMP1) to the tonoplast (TP) and the plasma membrane (PM). In *Arabidopsis* and tobacco two equally abundant DMP1 isoforms are synthesized by alternative translation initiation: a full length protein, DMP1.1, and a truncated one, DMP1.2, which lacks the N-terminal 19 amino acids including a TP-targeting dileucine motif. Accumulation of DMP1.1 and DMP1.2 in the TP and the PM, respectively, is Brefeldin A-sensitive, indicating transit via the Golgi. However, DMP1.2 interacts with DMP1.1, leading to extensive rerouting of DMP1.2 to the TP and “eclipsed” localization of DMP1.2 in the PM where it is barely visible by confocal laser scanning microscopy but clearly detectable by membrane fractionation. It is demonstrated that eGFP fusion to either DMP1 terminus can cause mistargeting artifacts: C-terminal fusion to DMP1.1 or DMP1.2 results in altered ER export and N-terminal fusion to DMP1.1 causes mistargeting to the PM, presumably by masking of the TP targeting signal. These results illustrate how the interplay of alternative translation initiation, presence or absence of targeting information and rerouting due to protein-protein interaction determines the ultimate distribution of a transmembrane protein between two membranes.

## Introduction

DUF679 membrane proteins (DMPs) are small integral membrane proteins with four predicted transmembrane domains. The ubiquitous occurrence of DMP proteins in green plants and their absence from other kingdoms indicate a role in plant-specific processes, but their biological functions are not known yet. DMPs do not share any sequence similarity to known channels, transporters or other membrane proteins in any kingdom. In *Arabidopsis thaliana* the *DMP* gene family consists of ten members which display distinct tissue- and development-specific expression patterns [1]. *DMP1* is highly up-regulated in senescing tissues and in dehiscence and abscission zones of siliques, but it is also expressed in other tissues [1, 2].

Since the first report of a plant protein targeted to two different subcompartments [3], the record of dual-targeted proteins, mostly soluble proteins, has increased to approximately 160 in *Arabidopsis* and more than 90 in other plants [4, 5]. The majority of dual-localized proteins, roughly 110 in *Arabidopsis*, are targeted to mitochondria and chloroplasts, where most of them are associated with prokaryote-type processes, for example DNA replication, recombination and repair, nucleotide metabolism, tRNA biogenesis and translation. However, targeting to mitochondria or chloroplasts and peroxisomes, nucleus or cytoplasm as the other compartment has also been observed [6–13]. Most dual-targeted proteins known to date are found in non-secretory compartments, but concomitant traffic of proteins to a non-secretory and a secretory compartment such as the ER, Golgi, vacuole, plasma membrane or the cell wall [14–22] and to different compartments of the secretory pathway have also been reported [23–25].

Based on N-terminal ambiguous presequences, in land plants more than 400 proteins are predicted to be dual-targeted to mitochondria and plastids [26]. Yet, to prove dual-targeting is challenging or even impossible by standard techniques like protein-tagging with fluorescent markers. In case of a highly biased distribution of a dual-targeted protein between two compartments, the abundant major fraction can mask the minor fraction (“eclipsed distribution”; [27]). The detection of dual-targeted proteins can be further complicated by conditional regulated distribution between subcellular compartments, for example cell age- or cell type-dependent or in response to cellular signaling or changes in the extracellular conditions [28, 29].

The molecular mechanisms of dual protein-targeting in eukaryotes are manyfold. Frequently two protein isoforms are synthesized that differ by the presence or absence of a targeting sequence. Such isoforms may result from alternative transcription initiation at a single gene, partial mRNA splicing or alternative translation initiation. Other mechanisms are the presence of competitive targeting signals on the same polypeptide, ambiguous signals, partial inaccessibility of signals due to protein folding, modification or blocking by an interacting protein, and partial or reverse translocation from organelles (reviewed in [28, 30, 31, 32]).

Here, we investigated dual-targeting of *Arabidopsis* DMP1, a transmembrane protein of the secretory pathway, to the tonoplast (TP) and the plasma membrane (PM). We show that by alternative translation inititation at two in-frame AUG codons of the *DMP1* transcript two protein products are generated in approximately equimolar ratio. The full length isoform DMP1.1 contains the TP-targeting information which is lacking in the truncated isoform DMP1.2. However, when coexpressed, DMP1.2 is largely rerouted to the TP by protein-protein interaction with DMP1.1, leading to a highly biased protein distribution between these two membranes. The occurrence of the DMP1.2 fraction in the PM, which is almost undetectable by live-cell imaging using DMP1 fusions with eGFP, was revealed by membrane fractionation, confirming “eclipsed” DMP1 protein distribution. We also demonstrate that the widely used eGFP fluorophore, when fused to the N- or the C-terminus of DMP1, leads to mistargeting or artifactual retention, exemplifying the need for independent methods when assigning the subcellular localization of transmembrane proteins.

## Results

### Two DMP1 protein isoforms are expressed in *Arabidopsis*

**Fig 1:**
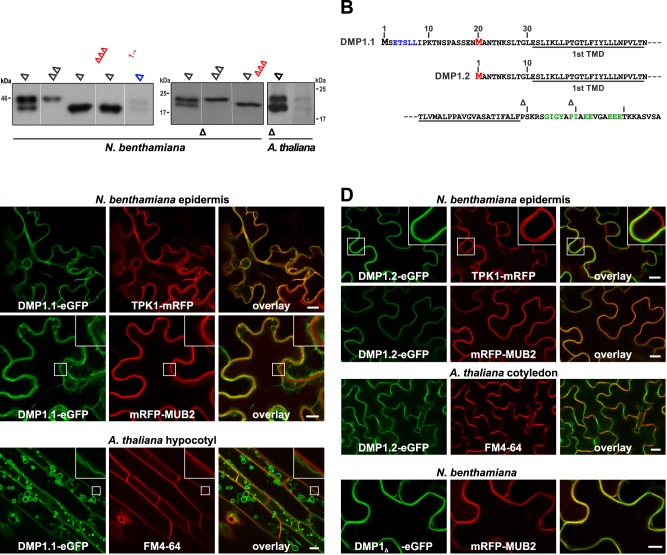
Identification and subcellular localization of DMP1 isoforms DMP1.1 and DMP1.2. (**A**) Western blot analysis of native and mutant DMP1 proteins transiently expressed in *Nicotiana benthamiana* abaxial leaf epidermis cells, in leaves of a transgenic *Arabidopsis thaliana* DMP1 overexpressor line, and in senescing leaves of wild-type *A*. *thaliana* Col-0 plants. Substitutions and deletions of the DMP1 open reading frame resulting in loss of the larger or the smaller isoform are indicated in red characters. Proteins were expressed from the 35S promoter (black characters) or the native *Arabidopsis DMP1* promoter (blue characters; ~20-times more protein was applied than in the other lanes). (**B**) Amino acid sequence of the DMP1.1 N-terminus with the second methionine in position 20 highlighted in red and a putative TP-targeting dileucine signal marked in blue letters (top line), the DMP1.2 N-terminus (center line), and the common C-terminus with putative ER-export signals highlighted in green letters (bottom line). TMD, transmembrane domain. (**C-E**) Determination by CLSM of (C) DMP1.1-eGFP, (D) DMP1.2-eGFP and (E) DMP1_ΔL6L7_-eGFP subcellular localization in coexpression experiments in transiently transfected tobacco abaxial epidermis cells (2 dpi) and transgenic *Arabidopsis* plants. The TP-located fusion protein TPK1-mRFP was used as TP marker and the PM-associated fusion protein mRFP-MUB2 as PM marker in tobacco. Staining of the PM in *Arabidopsis* plants was performed by incubating the cells for 10–15 min with the fluorescent dye FM4-64. Enlarged details in insets. Scale bars: 10 μm.

By protein blot analysis of tobacco epidermis cells overexpressing native *DMP1* or a *DMP1-eGFP* fusion construct, two proteins differing by approximately 2 kDa in mass in roughly equimolar ratio are detected by anti-DMP1 or anti-GFP antisera, respectively (Fig 1A), raising the question whether the two products result from post-translational processing or rather from alternative translation initiation. The DMP1 polypeptide contains in position 20 a methionine which, if used for translation initiation, would result in a 2 kDa shorter protein (Fig 1B). Substitution of this second methionine by alanine (M_20_A) both in DMP1-eGFP and untagged DMP1 leads to loss of the smaller proteins, whereas the deletion of the first 19 amino acids of DMP1-eGFP and DMP1 leads to loss of the respective larger proteins (Fig 1A). Mutating the M_1_ codon (ATG → CAC) results also in loss of the larger isoform. Thus, the M_20_ codon functions an alternative translation initiation codon giving rise to the 2 kDa smaller DMP1 isoform. We named the full length protein isoform DMP1.1 (22.1 kDa) and the truncated one DMP1.2 (20.1 kDa). Both DMP1 isoforms are also detected in tobacco cells transiently expressing DMP1-eGFP from the native *Arabidopsis DMP1* promoter and in *Arabidopsis* Col-0 wild type senescing leaves in which *DMP1* is highly up-regulated [1, 2], thus excluding that the occurrence of two protein isoforms is an overexpression artifact (Fig 1A).

### DMP1.1-eGFP and DMP1.2-eGFP are targeted to the TP and the PM, respectively

By using fluorescence imaging, we had previously observed that DMP1-eGFP localizes to the TP [1, 33]. We hypothesized that this localization may be accomplished by a putative TP-targeting signal (**E**TS**LL**) in the very end of the DMP1.1 N-terminus (Fig 1B). DMP1.2 lacks the first 19 amino acids (aa) containing this motif and thus should not be targeted to the TP. To address this hypothesis, we independently expressed DMP1_M20A_-eGFP (subsequently termed DMP1.1-eGFP) and DMP1_Δ1-19_-eGFP (termed DMP1.2-eGFP) in tobacco and *Arabidopsis* and analyzed their subcellular distribution in colocalization experiments. In transgenic *Arabidopsis* plants, DMP1.1-eGFP colocalizes with the TP-marker TPK1-mRFP but not with the PM-marker mRFP-MUB2 or the fluorescent dye FM4-64 (Fig 1C), demonstrating that DMP1.1-eGFP is targeted to the TP. In contrast, DMP1.2-eGFP does not colocalize with TPK1-mRFP but with mRFP-MUB2 and FM4-64, revealing that DMP1.2-eGFP is directed to the PM (Fig 1D). We further tested the functionality of the di-leucine based putative TP-targeting signal by deletion analysis. TP-targeting of DMP1_ΔL6L7_-eGFP is completely abolished and the protein accumulates exclusively in the PM (Fig 1E) like DMP1.2-eGFP. Thus, the LL dipeptide in the DMP1.1-eGFP N-terminus is critical for directing DMP1.1-eGFP to the TP. In its absence the PM appears to be the default membrane for DMP1 proteins.

**Fig 2:**
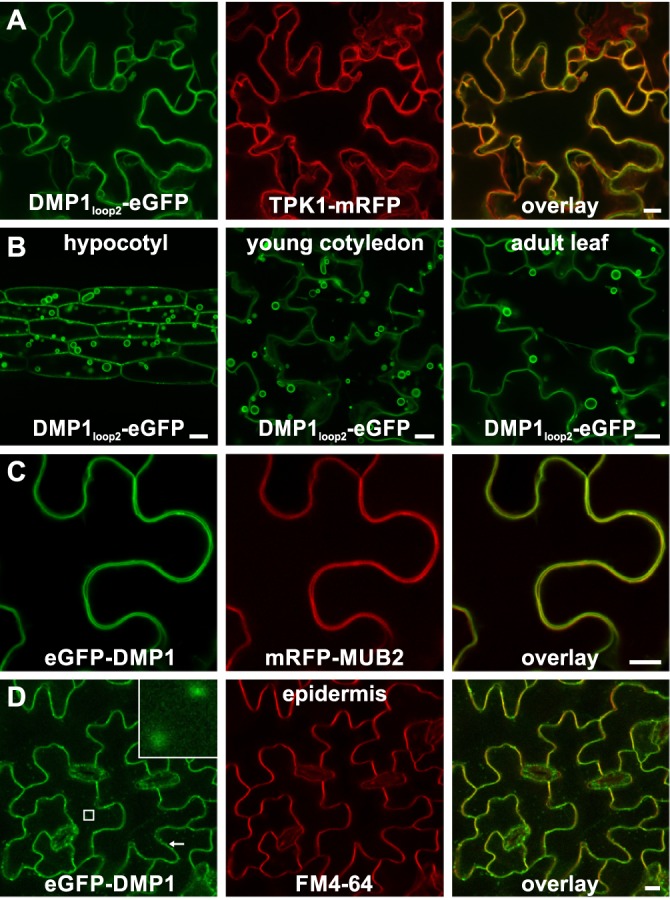
DMP1_loop2_-eGFP labels the TP and eGFP-DMP1 is mistargeted to the PM. Determination by CLSM of DMP1_loop2_-eGFP and eGFP-DMP1 subcellular localization. (**A**) DMP1_loop2_-eGFP colocalizes with TPK1-mRFP in the TP of tobacco epidermis cells. (**B**) In *Arabidopsis* hypocotyl, young cotyledon and adult leaves, DMP1_loop2_-eGFP localizes in the TP and vacuolar structures such as tonoplastic bulbs and transvacuolar strands but not in the ER membrane. (**C**) The N-terminal fusion protein eGFP-DMP1 colocalizes with mRFP-MUB2 in the PM of tobacco lower epidermis cells. (**D**) In *Arabidopsis* epidermis cells eGFP-DMP1 colocalizes with the fluorescent dye FM4-64 (10–15 min staining time) in the PM and additionally decorates endosomes (inset). Scale bars: 10 μm.

This conclusion was corroborated by the subcellular localization of the two proteins DMP1_loop2_-eGFP, which contains eGFP within the second loop (between transmembrane domains 2 and 3), and eGFP-DMP1, carrying an N-terminal eGFP fusion. Like DMP1.1-eGFP, DMP1_loop2_-eGFP is targeted to the TP in tobacco leaves (Fig 2A) and in *Arabidopsis* hypocotyl, cotyledon and leaves (Fig 2B). In contrast, eGFP-DMP1 is found in the PM in tobacco cells, where it colocalizes with mRFP-MUB2 (Fig 2C) and DMP1.2-mRFP (S1A Fig), and also in transgenic *Arabidopsis* epidermis cells, where it colocalizes with FM4-64 (Fig 2D). The mistargeting of eGFP-DMP1 to the PM presumably results from masking of the LL motif by eGFP, corroborating that the PM is the default target membrane for DMP1 proteins. Both in tobacco (S1B Fig) and *Arabidopsis* (Fig 2D, inset), minor eGFP-DMP1 amounts are found additionally in endosomes.

As had been described for DMP1-eGFP [33], depending on the tissue investigated, both DMP1.1-eGFP and DMP1.2-eGFP decorate to a certain extent the ER membrane in *Arabidopsis* (S2 Fig). Interestingly, DMP1_loop2_-eGFP and eGFP-DMP1 were never detectable in the ER. This apparent discrepancy will be addressed below.

### Trafficking of DMP1 isoforms is Golgi-dependent

We further investigated whether trafficking of DMP1.1 and DMP1.2 along the secretory pathway is Golgi-dependent. When tobacco epidermis cells that coexpress DMP1.1-eGFP or DMP1.2-eGFP and the Golgi marker Man49-mCherry are treated with Brefeldin A, both fusion proteins accumulate in the ER membrane (S3A and S3B Fig). Accordingly, trafficking of DMP1.1-eGFP and DMP1.2-eGFP to their final destinations TP and PM, respectively, is Golgi-dependent.

### DMP1.2-eGFP localization in the PM is suppressed by coexpression with DMP1.1-eGFP

**Fig 3:**
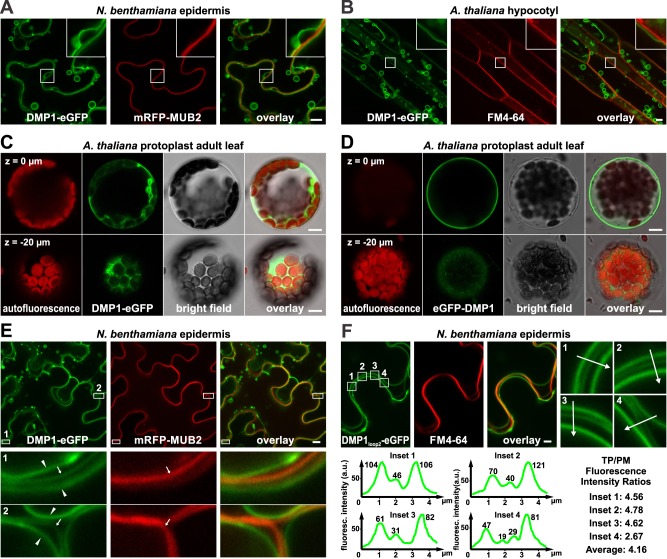
Labeling of the PM by DMP1-eGFP is mostly undetectable by CLSM. (**A**) Coexpression of DMP1-eGFP and PM-associated mRFP-MUB2 in tobacco lower epidermis cells and (**B**) expression of DMP1-eGFP in transgenic *Arabidopsis* cells that were briefly incubated (10–15 min) with FM4-64 to stain the PM shows segregation of fluorescence signals (enlarged details in insets). (**C**) Protoplasts were prepared from late adult *Arabidopsis* leaves expressing DMP1-eGFP or (**D**) eGFP-DMP1 for clear distinction between the fluorescence signals originating from the PM, the TP and the ER at this developmental stage. (**C** and **D**) Top rows show cross sections through the center plane of the protoplasts (z = 0 μm). DMP1-eGFP decorates the TP, whereas eGFP-DMP1 decorates the PM. Bottom rows show cross sections through the cortical region (z = -20 μm). They reveal labeling of endomembranes, presumably both the ER membrane and the TP only for DMP1-eGFP but not eGFP-DMP1. (**E**) Sporadically occurring DMP1-eGFP fluorescence signals at the PM in abaxial tobacco epidermis cells. DMP1-eGFP fluorescence signals from the plasma membranes of adjacent cells (arrows in insets 1 and 2) are weaker than those from the two vacuolar membranes (arrowheads in insets 1 and 2), indicating weaker accumulation of DMP1-eGFP in the PM compared to the TP. (**F**) Sporadical co-labeling of the TP and the PM was also observed with DMP1_loop2_-eGFP. Fluorescence intensity of labeled membranes was quantified along the 4.5 μm paths indicated by arrows in insets 1–4. TP versus PM fluorescence ratios (TP/PM) were calculated as the combined fluorescence values of the two TP signals divided by those of the two PM signals (merged in inset 1–3) for each cross section. Scale bars: 10 μm.

Interestingly, individually expressed DMP1.1-eGFP and DMP1.2-eGFP are directed exclusively to the TP and the PM, respectively (Fig 1C and 1D), whereas in earlier studies it was found that expression of DMP1-eGFP, which results in synthesis of DMP1.1-eGFP and DMP1.2-eGFP at approximately equimolar ratios (Fig 1A), labels only the TP (and the ER in old tissues [1, 33]), but not the PM. In a new assay the exclusive TP labeling by DMP1-eGFP in tobacco epidermis (Fig 3A) and *Arabidopsis* hypocotyl cells (Fig 3B) was confirmed. In both cell types DMP1-eGFP and the PM marker signals clearly segregate, indicating that in the presence of DMP1.1-eGFP, DMP1.2-eGFP does not localize to the PM at a detectable level.

In earlier experiments in old tissues (adult tobacco and senescing *Arabidopsis* leaves) DMP1-eGFP decorated besides the TP also the ER [1, 33]. To achieve a higher resolution and sensitivity, we investigated DMP1-eGFP localization in protoplasts from adult *Arabidopsis* leaves. DMP1-eGFP labels both the TP (Fig 3C, top row) and the ER (Fig 3C, bottom row). In contrast, eGFP-DMP1 localizes only in the PM (Fig 3D) like in intact tobacco and *Arabidopsis* cells (Fig 2C and 2D; S1A Fig). Sporadically, in individual cells minor amounts of DMP1-eGFP and DMP1_loop2_-eGFP fusion proteins were observed at the PM (Fig 3E and 3F), suggesting the occurrence of a marginal, by confocal laser scanning microscopy (CLSM) hardly detectable fraction in the PM.

To exclude artifacts due to ectopic overexpression of DMP1-eGFP, we analyzed the subcellular localization of DMP1-eGFP transiently expressed by the native *Arabidopsis DMP1* promoter in tobacco leaves and in primary roots of stably transformed *Arabidopsis* plants (S4 Fig). Also when only weakly expressed (see Fig 1A) in tobacco epidermis cells, DMP1-eGFP accumulates in the TP and clearly segregates from the mRFP-MUB2 stained PM (S4A Fig), resembling the distribution observed with the 35S promoter (Fig 3A). In roots of transgenic *Arabidopsis* plants, DMP1-eGFP is also associated with the TP. It decorates here multiple small vacuoles in the rapidly expanding cells of the transition zone that are fusing to form the central vacuole (S4B Fig). Interestingly, in non-elongated cells of the transition zone, but not in elongating cells, signals originating from the PM were visible, indicating occasional cell-specific labeling of the PM by DMP1-eGFP.

### DMP1.2 subcellular distribution in the PM and endomembranes

**Fig 4:**
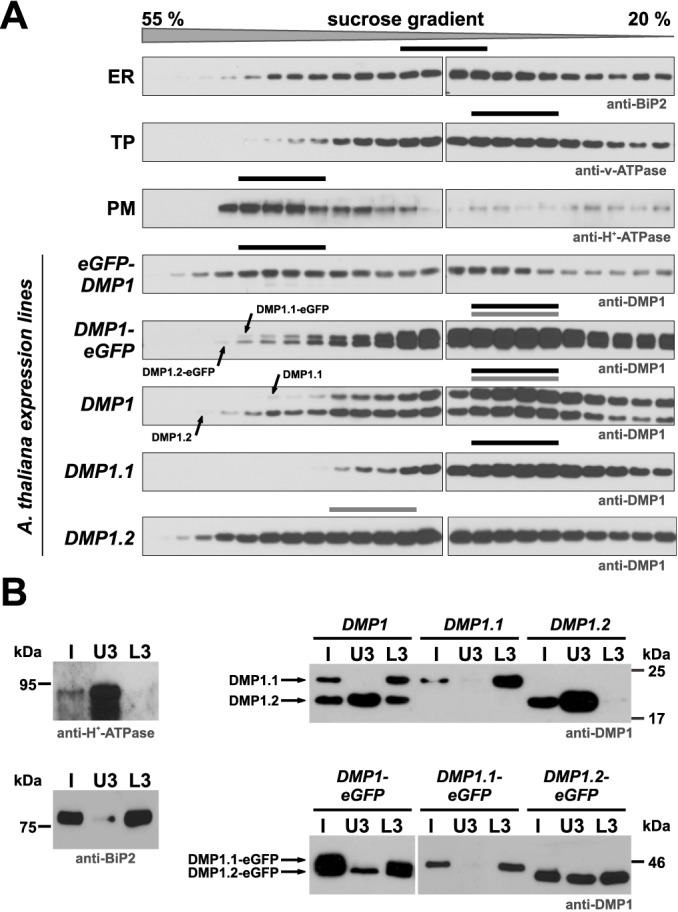
Distribution of DMP1 isoforms in fractionated membranes from different transgenic *Arabidopsis* DMP1 expression lines. (**A**) Microsomal membranes from 8 days old *DMP1-eGFP*, *eGFP-DMP1*, *DMP1*, *DMP1*.*1* and *DMP1*.*2* seedlings were fractionated on linear sucrose gradients and 10 μl of each fraction were analysed by Western blotting. Marker protein-specific antibodies (indicated below the three top panels) were used to visualize the distribution of microsomes originating from the ER, TP and PM. Anti-BiP2 detects the ER-resident protein BiP2, anti-v-ATPase detects tonoplast-derived vesicles and anti-H^+^-ATPase detects PM-derived vesicles. Depicted are representative distributions observed in all lines. Anti-DMP1 antibodies were used to detect all DMP1 isoforms and fusion proteins in the transgenic plants (five bottom panels). (**B**) Microsomal membranes from 8 days old *DMP1*, *DMP1*.*1*, *DMP1*.*2*, *DMP1-eGFP*, *DMP1*.*1-eGFP* and *DMP1*.*2-eGFP* seedlings were separated using an aqueous two-phase system. The PM-enriched upper phases U3 and endomembrane-enriched lower phases L3 were extracted three times to maximize PM and endomembranes separation. Separation of PM vesicles (U3) from endomembranes (L3) was monitored against the initial, unfractionated microsomal fraction (I) on Western Blots using anti-H^+^-ATPase and anti-BiP2 antibodies in each line (left panels). DMP1 proteins were detected using anti-DMP1 antibodies (right panels). 1 μg total protein was loaded for each fraction.

The almost exclusive subcellular localization of the DMP1-eGFP proteins in the TP appears to conflict with the PM localization of individually expressed DMP1.2-eGFP. We therefore scrutinized the DMP1 isoform distribution in transgenic *Arabidopsis* lines by fractionation of microsomes on linear sucrose gradients from 8 days old seedlings expressing eGFP-DMP1, DMP1-eGFP, DMP1, DMP1.1 or DMP1.2 (Fig 4A). The distribution of microsomes originating from the ER, the TP and the PM was monitored by using antibodies directed against proteins which are located exclusively in one of these compartments. The distributions of eGFP-DMP1 and DMP1-eGFP approximate the PM and TP markers, confirming the localizations observed by CLSM. Notably, the DMP1.1-eGFP and DMP1.2-eGFP proteins in the *DMP1-eGFP* transgenic plants display the same enrichment pattern in the lighter fractions, whereas in the denser fractions DMP1.2-eGFP is enriched relative to DMP1.1-eGFP. This may be explained by the presence of small DMP1.2-eGFP amounts in the PM (Fig 3E and 3F). In the transgenic *DMP1* plants, untagged DMP1.2 is even stronger enriched in the heavier fractions and correspondingly depleted in the less dense fractions relative to the DMP1.1 isoform. This suggests largely identical subcellular localization of DMP1.1 and DMP1.2, but DMP1.2 is additionally present in another membrane, presumably the PM. The DMP1.1 distribution in the *DMP1*.*1* line is comparable to that in *DMP1* plants. In contrast, the distribution of DMP1.2 in *DMP1*.*2* plants is conspicuously shifted towards the denser fractions compared to the same isoform in *DMP1* plants and closely resembles the patterns of eGFP-DMP1 and the PM marker. These results support the conclusion from colocalization experiments that, in the absence of DMP1.1, the predominant subcellular localization of DMP1.2 is the PM. The altered DMP1.2 distribution in the membrane fractions of *DMP1* plants indicates that the co-expressed DMP1.1 redirects DMP1.2 from the PM to the TP.

To determine DMP1 isoform distribution in an independent test, we purified PM vesicles from total microsomal fractions using an aqueous two-phase system (ATPS). Successful separation of PM vesicles from the endomembrane fraction, containing the TP- and ER-derived vesicles, was monitored using antibodies against PM-located H^+^-ATPase and ER-resident BiP2 as marker proteins (Fig 4B). In *DMP1*.*1* plants, DMP1.1 is detected exclusively in the lower fraction L3, consisting of enriched endomembranes, whereas DMP1.2 from *DMP1*.*2* plants occurs only in the upper U3 fraction enriched with PM vesicles. However, DMP1.2 is detected in both L3 and U3 fractions in *DMP1* plants, indicating that it is present both in the PM and in endomembranes, thus confirming the rerouting of DMP1.2 by DMP1.1. Interestingly, the C-terminal fusion of eGFP to DMP1 isoforms partially affects proper subcellular localization. DMP1.1-eGFP partitions, like untagged DMP1.1, solely to the endomembrane fraction L3. DMP1.2-eGFP is however, other than untagged DMP1.2, not found exclusively in the PM but both in the PM and endomembranes. DMP1.2-eGFP occurrence in the endomembrane fraction most likely originates from ER-derived vesicles because by CLSM it is detected in the PM and the ER but not in other endomembranes (Fig 1 and S2 Fig). In *DMP1-eGFP* plants, the DMP1.2-eGFP isoform is also found in both the PM and the endomembrane fractions, whereas in *DMP1* plants the DMP1.2 isoform appears to be enriched in the PM fraction (Fig 4B). This is presumably caused by additional ER retention, which would also explain why the PM-fraction was difficult to detect by CLSM. Thus, the ER localization observed for all DMP1 C-terminal fusions appears to be artifactual (see below).

### DMP1.2 is rerouted to the TP by protein-protein interaction with DMP1.1

**Fig 5:**
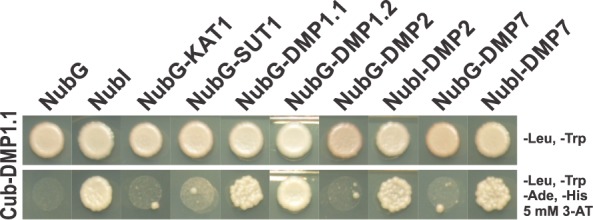
Analysis of DMP1 isoform interactions by the split-ubiquitin assay in yeast. DMP1.1-DMP1.1 and DMP1.1-DMP1.2 interactions were investigated using the split-ubiquitin system in yeast. Cub-DMP1.1 fusion protein was used as bait and appropriate growth and selection conditions were established using co-expression with NubG, NubG-KAT1, NubG-SUT1 (negative controls) and NubI (positive control). Cub-DMP1.1 interacts with NubG-DMP1.1 and NubG-DMP1.2, respectively, but not with the DMP1-homologs DMP2 or DMP7.

The modulation of DMP1.2 transit through the secretory pathway by DMP1.1 could be effected by protein-protein interaction between the isoforms. To test this hypothesis we analysed the ability of DMP1 isoforms to interact in the split-ubiquitin system in yeast (Fig 5). Cub-DMP1.1 interacts with NubG-DMP1.1, NubG-DMP1.2 and the positive control NubI (cytosolic), but neither with the negative controls NubG (cytosolic), NubG-KAT1 or NubG-SUT1 (transmembrane proteins) nor with NubG-DMP2 or NubG-DMP7. Accordingly, DMP1.1 is able to homodimerize and to form DMP1.1-DMP1.2 heterodimers, but it does not interact with the related DMP2 or DMP7 protein family members.

**Fig 6:**
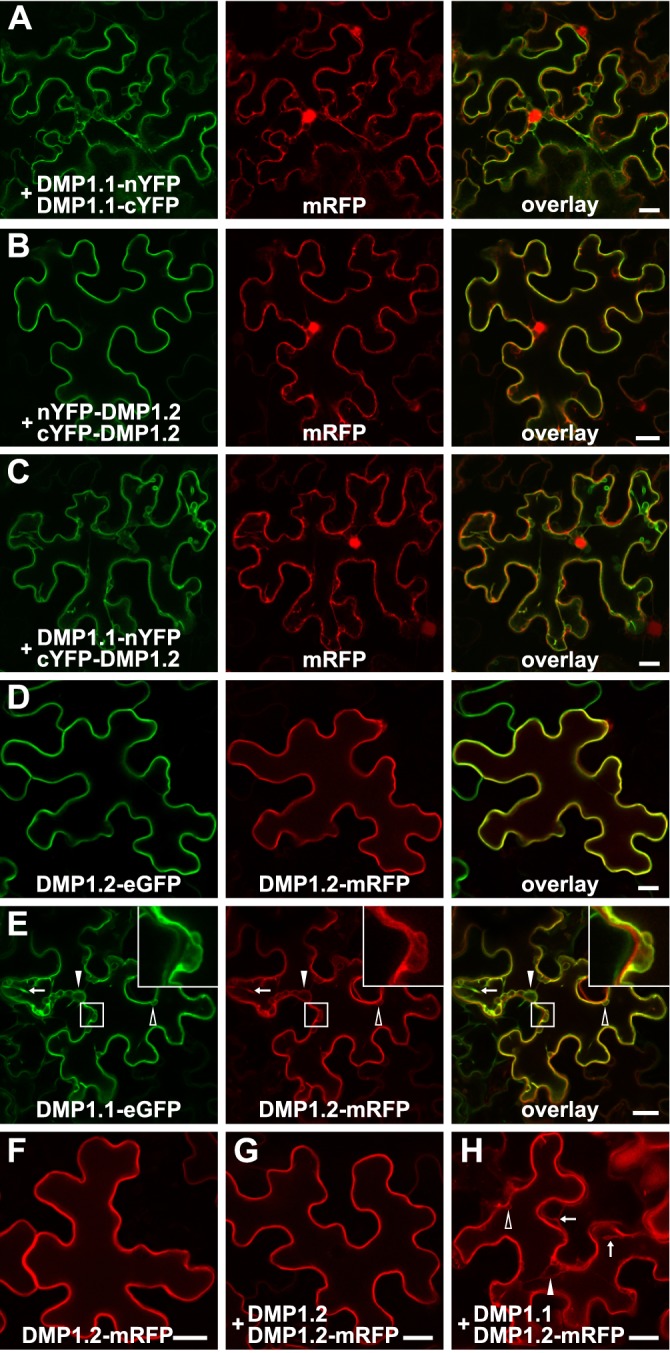
Rerouting of DMP1.2 to the TP by DMP1.1. (**A-C**) Left panels: Fluorescence signals of the indicated coexpressed fusion proteins containing the two YFP moieties (nYFP and cYFP) at 2 dpi in tobacco epidermis cells. Center panels: mRFP fluorescence signals visualizing the cytoplasm and the lumen of the nucleus. Right panels: superimposed YFP and mRFP signals. The three proteins for each assay were encoded on the same vector to ensure synchronized expression and equimolar protein levels. (**D-E**) DMP1.2-mRFP subcellular localization was investigated in coexpression experiments with (**D**) DMP1.2-eGFP and (**E**) DMP1.1-eGFP. The enlarged insets highlight residual DMP1.2-eGFP in the PM in presence of DMP1.1-eGFP. To exclude protein-protein interaction between the two fluorophores, fluorescence patterns of (**F**) DMP1.2-mRFP expressed individually, (**G**) DMP1.2-mRFP expressed in the presence of unfused DMP1.2, and (**H**) DMP1.2-mRFP expressed in the presence of unfused DMP1.1 were investigated. (**E**) and (**H**): Arrows indicate transvacuolar strands; filled arrowheads indicate the nucleus; open arrowheads indicate other TP-enclosed smaller organelles. Scale bars: 20 μm.

DMP1 isoform interaction was further detailed by ratiometric bifluorescence complementation (rBiFc) in transfected tobacco cells (Fig 6A–6C). Coexpression of DMP1.1-nYFP with DMP1.1-cYFP and nYFP-DMP1.2 with cYFP-DMP1.2 lead to strong fluorescence signals which can be clearly attributed to localizations in the TP (Fig 6A) or the PM (Fig 6B), respectively, matching the observations with DMP1.1-eGFP (Fig 1B) and DMP1.2-eGFP (Fig 1C). Coexpression of DMP1.1-nYFP with cYFP-DMP1.2 leads to distinct fluorescence signals in the TP, but not in the PM or other membranes (Fig 6C), demonstrating dimerization (or oligomerization) between DMP1.1 and DMP1.2 and as consequence rerouting of DMP1.2 to the TP. In the absence of protein-protein interaction, coexpression of DMP1.1-nYFP and cYFP-DMP1.2 would give rise to spatial separation of the proteins in the TP and PM, respectively, and consequently no fluorescence signals. These results demonstrate rerouting of DMP1.2 to the TP by interaction with DMP1.1, but they do not allow inferences on homodimerization of DMP1.2, as coexpression of DMP1.1-cYFP or cYFP-DMP1.2 with unfused nYFP also leads to fluorescence signals (S6E and S6J Fig) comparable to those obtained with DMP1.1-nYFP/DMP1.1-cYFP and nYFP-DMP1.2/cYFP-DMP1.2 (Fig 6A and 6B).

Additional colocalization experiments of DMP1.2-eGFP and DMP1.2-mRFP in tobacco revealed perfectly superimposed fluorescence signals in the PM (Fig 6D), whereas DMP1.1-eGFP and DMP1.2-mRFP coexpression signals are only partially congruent (Fig 6E). DMP1.1-eGFP decorates exclusively the TP and visualizes apparent transvacuolar strands (Fig 6E, left panel, arrow), the nucleus and organelles. DMP1.2-mRFP fluorescence signals overlap with DMP1.1-eGFP signals in the TP and vacuolar structures, but in contrast to DMP1.1-eGFP DMP1.2-mRFP additionally decorates the PM (Fig 6E, inset overlay). Thus, DMP1.2-mRFP is partially rerouted from the PM to the TP when coexpressed with DMP1.1-eGFP. To exclude artifacts due to the fluorophores in both fusion proteins, we expressed DMP1.2-mRFP individually (Fig 6F) and in the presence of untagged DMP1.2 (Fig 6G) or DMP1.1 (Fig 6H). Coexpression of DMP1.2 does not change the DMP1.2-mRFP localization in the PM (Fig 6G), while the presence of DMP1.1 leads to additional decoration of the TP (Fig 6H) like DMP1.1-eGFP (Fig 6E), confirming rerouting of DMP1.2-mRFP.

### ER retention of DMP1 C-terminal fusion proteins is artifactual

The membrane fractionation results (Fig 4B) suggest that ER localization of DMP1.2-eGFP is artifactual. ER retention was observed by CLSM when eGFP was fused to the C-terminus of DMP1.2, but not for eGFP-DMP1 or DMP1_loop2_-eGFP, which indicates that the eGFP tag fused at the DMP1 C-terminus is responsible for the mistargeting. Astoundingly, the degree of mistargeting is tissue age-dependent. In adult *Arabidopsis* rosette leaves DMP1.2-eGFP strongly labels the ER (S2 Fig), whereas in young cotyledons ER-labeling is only weak (Fig 1D). Similarly, in mature tissues such as older cotyledons or adult rosette leaves (S2 Fig), DMP1.1-eGFP fluorescence in the ER is strong, as had previously been observed for DMP1-eGFP [33], while in hypocotyls (Fig 1C) or young cotyledons (S2 Fig), ER-located DMP1.1-eGFP is not or only barely visible.

Erroneous ER localization of C-terminal DMP1 fusions could be explained by shielding of an ER export signal. Indeed, the DMP1 C-terminus contains three putative ER export signals, a PI dipetide and the acidic motifs EE and EEE (Fig 1B) [34–38]. We mutated these motifs in eGFP-DMP1, generating eGFP-DMP1_(P189A/I190A)_, eGFP-DMP1_(E192A/E193A)_ and eGFP-DMP1_(E197A/E198A/E199A)_. The mutant proteins still properly reach the PM, indicating functional ER export (S5A–S5C Fig), whereas the substitution of all five aspartate residues by alanine results in ER retention (S5D Fig), indicating that the C-terminus of DMP1 is indeed crucial for ER export. However, although other DMP members also exit the ER [1], DMP1 is the only member of the DMP protein family which contains these motifs. We then mutated a highly conserved domain in the C-terminus of DMP proteins (GIGY to AAAA, see Fig 1B) and found that this mutation also results in impaired ER export (S5E Fig). Hence, several amino acid motifs, or a conserved topology, in the DMP1 C-terminus are required for ER export, and these motifs are shielded by C-terminally fused eGFP in a tissue age-dependent manner.

### DMP1 topology

The split-ubiquitin (Fig 5) and rBiFc results (Fig 6A–6C) indicate that the DMP1.1 and DMP1.2 N- and C-termini face the cytosol. To substantiate the DMP1.1 and DMP1.2 topology, all possible combinations of N- and C-terminal nYFP/cYPF fusions to DMP1.1, DMP1.2 and DMP1.1+DMP1.2 were tested by rBiFc (S6 Fig). The results consistently confirmed that both N- and C-termini of DMP1.1 and DMP1.2 are cytosolic, and the association of the fluorescence signals observed for the three DMP1.1+DMP1.2 combinations with the TP (S6K–S6M Fig) corroborates that DMP1.2 is rerouted to the TP by protein-protein interaction with DMP1.1. In addition, DMP1 protein orientation was verified by protease treatment of eGFP-DMP1- or DMP1-eGFP-containing tobacco microsomes. From both fusion proteins free eGFP is released by mild proteinase K digestion (S7A Fig), indicating that eGFP faces the cytosol. The strong fluorescence signals of eGFP-DMP1 and DMP1-eGFP further supports this interpretation, because the acidic environment within the vacuolar lumen or the extracellular space would prohibit eGFP fluorescence. The strong signals obtained with DMP1_loop2_-eGFP demonstrate that the second loop is also facing the cytosol, confirming the predicted DMP1 topology with four TMDs.

The DMP1 cysteine residues C58 and C147 are located in the first and third loop (S7B Fig). We hypothesized that these residues may form a disulfide bond which would require that these two loops face the ER lumen where the protein disulfide isomerases predominantly reside [39]. We generated DMP1_C58A/C147A_-eGFP and investigated if these mutations have an impact of DMP1 subcellular localization (S7C Fig). The mutated fusion protein is largely retained in endomembranes, strongly labeling the ER network and associated aggregates of various sizes. Thus, accurate protein folding or maintenance of DMP1 conformation probably involves the formation of a disulfide bridge, indicating a role of protein folding in proper targeting of DMP1 and confirming DMP1 topology.

## Discussion

### Alternative translation initiation at *DMP1* transcripts leads to two differentially targeted DMP1 protein isoforms

**Fig 7:**
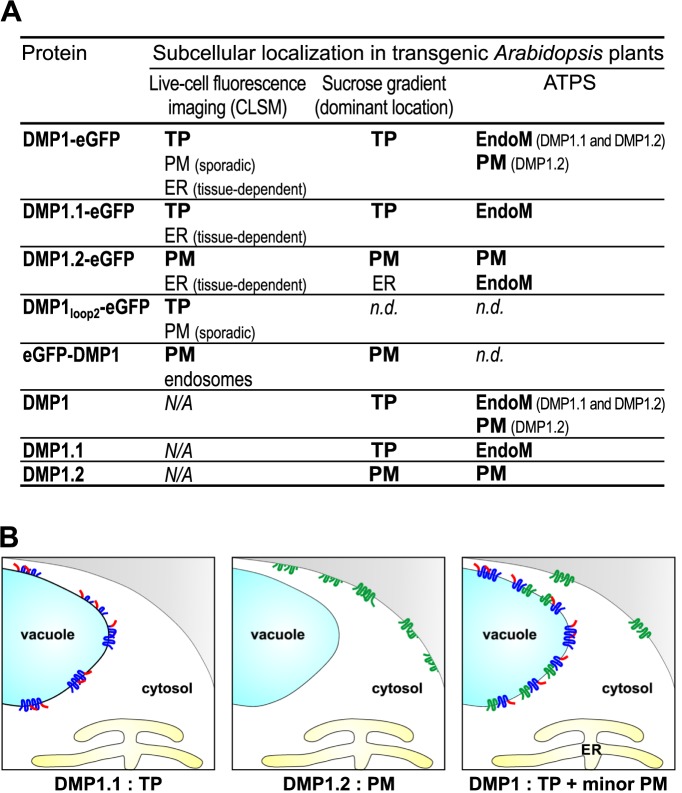
Overview and model. (**A**) Summary of subcellular location results. (**B**) Model for the localization of DMP1.1 and DMP1.2 proteins when individually or co-expressed. Individually expressed DMP1.1 is targeted to the TP and DMP1.2 to the PM. DMP1.2 is efficiently rerouted to the TP upon protein-protein interaction with DMP1.1 and only a minor fraction is targeted to the PM. Additional marginal accumulation of DMP1.1-eGFP and DMP1.2-eGFP in the ER membrane observed in some tissues is artifactual and not depicted. As both DMP1.1 and DMP1.2 trafficking to the TP or the PM is Brefeldin A-sensitive, DMP1.1-DMP1.2 protein-protein interaction presumably takes place in the ER membrane, Golgi and/or prevacuolar compartments before transit to either the TP or the PM.

In this study we have demonstrated that the *Arabidopsis DMP1* transcript is, probably due to “leaky ribosome scanning” [40], translated into two approximately equally abundant membrane protein isoforms, DMP1.1 (22.1 kDa) and DMP1.2 (20.1 kDa), which are targeted to the TP and the PM, respectively. We showed that DMP1.2, although lacking a targeting signal, is found in both the PM and the TP, due to partial rerouting by physical interaction with DMP1.1. Trafficking of DMP1.1 to the TP and of DMP1.2 to the PM were both shown to be Golgi-dependent. Fig 7 summarizes the subcellular location results and depicts a model for the localization of DMP1.1 and DMP1.2 proteins when individually or co-expressed.

Alternative translation initiation at the *DMP1* transcript, leading to synthesis of two differentially targeted protein isoforms, is reminiscent of dual-targeting of proteins of the non-secretory pathway such as organellar DNA polymerases POLγ1 and POLγ2 to mitochondria and chloroplasts [41, 42], holocarboxylase HCS1 to mitochondria and the cytosol [43] or yeast MOD5 to mitochondria, cytosol and the nucleus [44]. Only few dual-targeted plant membrane proteins of the secretory pathway have been reported. Several members of the SNARE family have dual or multiple locations in the secretory pathway [23, 24], and the vacuolar sorting receptors [22], the pyrophosphatase in cauliflower inflorescences [21] and the tonoplast intrinsic proteins TIP3;1 and TIP3;2 [25] were found in both the TP and the PM. The mechanisms leading to these dual distributions are unknown, though.

### DMP1.1 requires a TP targeting and an ER export signal for proper trafficking to its final destination

The differential targeting of DMP1.1 and DMP1.2 to the TP and the PM, respectively, depends on the presence of the dileucine motif **E**TS**LL** in DMP1.1 and its absence in DMP1.2. In recent studies, dileucine based motifs ((D/E)X_3-5_L(L/I)) [45] have been shown to be required in *Arabidopsis* for sorting of various proteins to the TP: the inositol transporter INT1 [46], members of the NITRATE TRANSPORTER 1/PEPTIDE TRANSPORTER (NPF) family (NPF8.3/PTR2/NTR1, NPF8.4/PTR4 and NPF8.5/PTR6; [47]), the monosaccharide transporter ESL1 [48], the two-pore calcium channel TPC1 [49], the molybdate carrier MOT2 [50] and the iron transporter VIT1 [51]. The fusion of dileucine motifs from INT1, NPF8.3 and NPF8.5 to the unrelated PM-located protein SUC2 caused rerouting of SUC2 to the TP [46, 47], indicating that TP-targeting is dominant over PM-targeting. Except for NPF8.3, which was retained in endomembranes, deletions or mutations of the dileucine motifs of INT1, ESL1, TPC1, MOT2 and VIT1 redirected the mutated proteins to the PM. Thus, although it remains a matter of debate whether in plants the PM is the default pathway in intracellular trafficking for all membrane proteins, it clearly is for DMP1 proteins.

Targeting of many membrane proteins to their final destination in the secretory pathway requires a cytosolic ER export signal in addition to the targeting information [38]. After synthesis at the ER membrane, cargo membrane proteins are incorporated into coat protein complex II (COPII) vesicles [52] upon interaction between coat proteins and ER export signals before delivery to the Golgi [53, 54]. Various export signals have been described in yeast, mammals and plants, including diacidic (D/E-X-D/E), dihydrophobic (ISI), diaromatic motifs (FF, YY and FY) [35, 37, 38, 51, 55, 56] and the motif PI [36]. Impaired or altered recognition of export signals due to mutations or masking by fusion moieties may result in partial or total retention of the protein within the ER membrane. The tonoplast-located *Arabidopsis* iron transporter VIT1 [57] contains both a LL-motif and an ER export signal in its N-terminus [51]. Mutation of the LL-motif led to rerouting to the PM whereas mutation of the dihydrophobic tripeptide **I**S**I** led to retention in the ER. Similar to DMP1.1, VIT1 requires two functional signals for proper targeting to its final destination, the TP. For DMP1 we have demonstrated that the ER retention of C-terminal fusions is artifactual, suggesting that ER export information is contained within the DMP1 C-terminus. Mutation of the C-terminal motifs PI, EE and EEE did not affect ER export of eGFP-DMP1, though, but only mutating both EE and EEE motifs together or mutating the conserved GIGY motif resulted in ER retention. Thus, it remains unclear if both regions act synergistically as ER export signals or if the mutation of one region affects the recognition of the ER export site by altering DMP1 C-terminus conformation. The dependence of proper ER export on accurate conformation of multiple DMP1 domains is highlighted by retention of DMP1 in the ER membrane when the cysteine residues in the first and third loop are mutated, presumably impairing the formation of a disulfide bridge. These two cysteine residues are conserved in all ten DMPs [1], suggesting a crucial structural role in all DMP members.

### Mislocation of DMP1 fusion proteins due to positional effects of eGFP and DMP1.2 “eclipsed” distribution

The inconsistent localization of the different fusion proteins in *Arabidopsis* (summarized in Fig 7A) and tobacco highlights the risk of misinterpreting subcellular localization results obtained by fusing proteins with bulky fluorescent tags. We have shown for DMP1.1 that N-terminal eGFP fusion causes mistargeting to the PM, whereas DMP1.1 and DMP1.2 with C-terminal eGFP fusions exhibit artifactual ER retention. The mistargeting of eGFP-DMP1 likely results from masking of the dileucine motif and subsequent transit to the PM. Mistargeting of fusion proteins due to masking of signal sequences appears to be not uncommon. It has been reported for several proteins such as the Golgi-located endomembrane protein EMP12 that is mislocated to post-Golgi compartments upon masking of its Golgi retention signal by eGFP [58]. However, for DMP1 proteins the PM is not an entirely abnormal location, because DMP1.2 is partially targeted to this membrane. This may indicate a role for DMP1 in both the TP and the PM. Yet, in the presence of DMP1.1 the minor DMP1.2 fraction that is naturally targeted to the PM, is almost completely “eclipsed” and not detectable by CLSM. Only by membrane fractionation approaches it was possible to decipher the complex subcellular distribution of DMP1 isoforms and to reveal protein mislocation due to eGFP fusion tags.

### Rerouting of DMP1.2 to the TP by interaction with DMP1.1

DMP1.2 is rerouted to the TP upon direct interaction with DMP1.1. A comparable retargeting mechanism had been discovered for the maize ZmPIP1 aquaporines [59]. Expressed individually, ZmPIP1 is retained in the ER membrane, but when co-expressed with ZmPIP2 it is relocalized to the PM through ZmPIP1-ZmPIP2 heterooligomerization. Relocalization of proteins from the ER or Golgi to the PM due to heterooligomerization has also been reported for other receptors or channels in mammals and plants [60–62].

Though DMP1.1 homodimerization events were detected by the split-ubiquitin system, the split-YFP system used was not sensitive enough. The use of free nYFP as negative control led to strong signals when co-expressed with any DMP1.1 or DMP1.2 proteins fused to cYFP, independently of the orientation (C- or N-terminal fusions). Indeed, the split-YFP system bears a risk of occasional reassembly of the two YFP halves in the absence of interactions between the fused proteins and dimerization of YFP [63, 64]. However, in spite of the low signal-to-noise ratio of the system, the detected interaction between DMP1.1 and DMP1.2 is credible as the fluorescence signals can be clearly attributed to the TP and are independent of the fusion protein combinations (NN, NC, CN and CC), with no signals originating from the PM.

Localization studies using eGFP fusion proteins and proteomic approaches have revealed that several membrane protein families include both TP- and PM-located proteins, among them members of the INT, NPF and aquaporine families [39, 65–80]. Although the substrate specificities of transporters or carriers belonging to the same protein family can differ, they may fulfil comparable functions at both membranes. For example, Wolfenstetter *et al*. [46] could show that the PM-located INT4 is functionally active in the TP by successfully rescuing an *int1 Arabidopsis* mutant, which lacks the TP-located INT1, with a TP-rerouted INT4 version. The subcellular localization of the *Arabidopsis* DMP1 proteins reported here is a crucial step towards unravelling the biological activities of DMP1.1 and DMP1.2 and elucidating whether DMP1.2 fulfills the same function in both the TP and the PM.

## Materials and methods

### Plasmid constructs

All PCR reactions were performed using Phusion® High-Fidelity DNA Polymerase (New England Biolabs, Frankfurt, Germany). The integrity of PCR products was verified by sequencing. Generation of *35*:*DMP1-eGFP*, *DMP1p*:*DMP1-eGFP*, *35S*:*TPK1-mRFP* and *35S*:*mRFP-MUB2* constructs was described previously [1, 33]. Primers, vectors, templates and cloning strategies used to generate protein expression constructs are listed in S1 Table. Destination vector pB2GW7 is described in Karimi et al. [81]. pBT3-N was purchased from Dualsystems Biotech AG (Schlieren, Switzerland). NWTX_GW and NX32_GW were obtained from ABRC (https://abrc.osu.edu). The destination vectors pBiFCt-2in1-NN, pBiFCt-2in1-NC, pBiFCt-2in1-CN and pBiFCt-2in1-CC are described by Grefen and Blatt [82]. The Golgi-marker Man49-mCherry is described by Nelson et al. [83] and was obtained from ABRC.

### Plant material, growth conditions and plant transformation

*Arabidopsis thaliana* Col-0 and *Nicotiana benthamiana* plants were grown and stably or transiently transformed as described previously [1]. *Agrobacterium tumefaciens* cultures were resuspended at OD_600nm_ = 0.05 in infiltration solution [84] prior to tobacco infiltration. Subcellular localization of fusion proteins transiently expressed in tobacco epidermis cells was investigated two days post infiltration (dpi). To stain the plasma membrane in cells from *Arabidopsis* hypocotyls, cotyledons or adult leaves the tissue samples were submerged for 10–15 min in water containing 10 μM FM4-64 (SynaptoRed^TM^, Sigma-Adrich).

### Fast protoplast isolation

*Arabidopsis* leaves were cut into stripes with a razor blade in a drop of 0.5 M mannitol and incubated for 1 h to allow plasmolysis. The solution was replaced by the enzyme mixture consisting of 0.4 M mannitol, 8 mM CaCl_2_, 1% cellulase and 0.25% Macerozyme R-10, pH 5.5. Samples were shaken overnight in darkness at 20 rpm before analysis.

### Live-cell imaging using confocal laser scanning microscopy (CLSM)

CLSM was performed on a Leica TCS-SP5 AOBS (acousto-optical beam splitter) confocal laser scanning microscope equipped with water immersion objectives. Excitation/emission wavelengths used for multi-colour imaging of cells co-expressing proteins fused to eGFP and proteins fused to mRFP or mCherry were 488 nm/495 nm—530 nm for eGFP and 561 nm/585 nm—655 nm for mRFP and mCherry. In case of crosstalk, imaging was performed by sequential scanning. Simultaneous excitation of eGFP and FM4-64 was performed using the 488 nm line of the argon laser and emission bandwidths 495 nm—550 nm for eGFP and 600 nm—660 nm for FM4-64. Simultaneous excitation of YFP and mRFP in rBiFc assays was performed using the 514 nm line of the argon laser and the 561 nm line of the diode-pumped solid state laser, respectively. YFP and mRFP emissions were recorded using the bandwidths 520 nm—545 nm and 585 nm—655 nm, respectively. Post-acquisition image processing and fluorescence quantification was performed using the Leica LAS AF software.

### Western blotting and antibodies

10 μl of membrane fractions were denaturated at 95°C for 10 min in Laemmli buffer, size fractionated by SDS/PAGE, transferred to PVDF membranes (Immobilon-P; Merck Millipore, Darmstadt, Germany) by semi-dry blotting and detected by chemiluminescence (Pierce^TM^ ECL Western Blotting Substrate, Thermo Fisher Scientific Inc.).

Membranes were incubated for 1.5 h with the appropriate antibody diluted according to manufacturer's instructions in TBST containing 6.5% non-fat dry milk. Membranes were washed three times for 5 min with TBST and, when required, incubated with the secondary antibody for another 1.5 h. Primary antibodies used were rabbit anti-GFP-HRP (Santa Cruz Biotechnology, Inc.), rabbit anti-DMP1 (Thermo Fisher Scientific Inc.), rabbit anti-BiP2 (Agrisera) for detection of ER-microsomes, rabbit anti-H^+^-ATPase (Agrisera) for detection of PM-derived vesicles and rabbit anti-v-ATPase (Agrisera) for detection of tonoplast-derived vesicles. Goat anti-mouse or anti-rabbit IgG-HRP (Santa Cruz Biotechnology, Inc) were used as secondary antibodies. Custom anti-DMP1 primary antibodies were raised against the DMP1 C-terminal peptide KRSGIGYAPIAEEVGAE corresponding to aa 181 to 197 of DMP1.1. The unpurified rabbit anti-serum was used (1:5,000 dilution) for detection of unfused DMP1 proteins.

### Proteinase K treatment of microsomes

Tobacco leaves transiently expressing DMP1-eGFP, eGFP-DMP1 or eGFP were homogenized in extraction buffer without detergent. The resulting microsomes were pelleted by ultracentrifugation and resuspended in buffer without detergent, followed by Proteinase K (Carl Roth, Karlsruhe, Germany) treatment using 0.1 mg/ml proteinase K for 15 min at 37°C. Detection by Western blotting was performed using anti-GFP and anti-DMP1 antibodies.

### Membrane fractionation on continuous sucrose gradient

*Arabidopsis thaliana* seedlings were grown in liquid ½ MS medium supplemented with 1% sucrose and 0.5 g MES for seven days. 13 g plant material were homogenized using pestle and mortar in 38 ml buffer containing 50 mM Tris (pH 7.6 at 22°C), 150 mM NaCl, 20% glycerol, 1 mM PMSF, 2 mM EDTA and 1 X Halt Protease inhibitor cocktail (Thermo Scientific). Lysates were centrifuged for 5 min at 4,000 rpm (4°C). Supernatants were filtered through two layers of Miracloth and centrifuged for 5 min at 4,000 rpm (4°C). Subsequently supernatants were centrifuged for 45 min at 100,000 g (4°C). The resulting microsomal pellets were resuspended thoroughly by pipetting in 625 μl phase buffer containing 10 mM Tris (pH 7.6 at 22°C), 10% sucrose, 1 mM DTT and 2 mM EDTA. Samples were centrifuged for 5 min 4,000 rpm (4°C). 650 μl of supernatant were loaded on continuous sucrose gradient and allowed to separate for 16 h at 100,000 g (4°C). Fractions of approximately 300 μl were collected using a peristaltic pump. The 20% - 55% continuous sucrose gradients were prepared by layering successively 500 μl sucrose solutions of decreasing sucrose concentrations (55%, 50%, 45%, 40%, 35%, 30%, 25% and 20%) which were allowed to diffuse for 8 hours at 4°C.

### Isolation of plant plasma membranes by aqueous polymer two-phase partitioning (ATPS)

*Arabidopsis thaliana* seedlings were grown in liquid ½ MS medium supplemented with 1% sucrose and 0.5 g/l MES for seven days. 10 g plant material were homogenized using pestle and mortar in 30 ml extraction buffer containing 0.3 M sucrose, 50 mM MOPS-KOH, 5 mM Na-EDTA pH 7.5, 1 mM PMSF, 5 mM DTT, 5 mM insoluble PVPP, 5 mM ascorbic acid and 1 X Halt Protease inhibitor cocktail. Lysates were centrifuged for 5 min at 4,000 rpm (4°C). Supernatants were filtered through two layers of Miracloth and centrifuged for 5 min at 4,000 rpm (4°C). Subsequently supernatants were centrifuged for 45 min at 100,000 g (4°C). The resulting microsomal pellets were resuspended thoroughly in 9 ml resuspension buffer 1 (0.3 M sucrose, 5 mM K-Phosphate buffer pH 7.8, 0.1 mM Na-EDTA and 1 mM DTT) and centrifuged for 5 min at 4,000 rpm (4°C). 9 g microsomal fraction was added to 27 g phase solution consisting of precooled 11.52 g Dextran 20% (Dextran 500000), 5.76 g PEG 40% (PEG 3350) and 9.72 g phase buffer (0.33 M sucrose, 5 mM K-Phosphate buffer pH 7.8, 3 mM KCl), mixed by inverting 25 times. Phase separation was performed by centrifugation for 10 min at 1,500 g (4°C) in a swing-out rotor giving rise to a turbid PEG-enriched upper phase (U1) containing purified PM-vesicles and a green Dextran-enriched lower phase (L1) containing purified endomembranes. In parallel, tubes containing 36 g phase solution were prepared (11.52 g Dextran 20% (Dextran 500000), 5.76 g PEG 40% (PEG 3350) and 18,72 g phase buffer), mixed 25 times and also centrifuged for 10 min at 1,500 g (4°C) in a swing-out rotor to obtain pure upper and lower phases. U1 and L1 were further purified in two successive wash steps with pure lower and upper phases, giving rise to U3 and L3, respectively. Both U3 and L3 were diluted at least 5 times with resuspension buffer 1 and membranes were pelleted by ultracentrifugation (45 min at 100,000 g and 4°C). Pellets were dissolved in resuspension buffer 2 (10% sucrose, 25 mM Hepes pH 6.6, 5 mM EDTA and 1 X Halt protease cocktail inhibitor) and protein concentration was determined by BCA assay.

### Protein-protein interaction studies by ratiometric bifluorescence complementation assay (rBiFc)

Constructs were transiently introduced into tobacco abaxial epidermis cells by *Agrobacterium tumefaciens*-mediated transformation. rBiFc assays were performed at 2 dpi by CLSM.

### Protein-protein interaction studies using the yeast split-ubiquitin system

Yeast strain THY.AP4 (*MATa*, *ura3*, *leu2*, *lexA*::*lacZ*::*trp1*, *lexA*::*HIS3*, *lexA*::*ADE2*) was cotransformed with both bait and prey vectors by the LiOAc/ssDNA/PEG method [85] and positive colonies were selected for 2 days at 28°C on SD medium lacking leucine and tryptophane. Protein-protein interaction was detected by 5 days growth on SD medium additionally lacking adenine and histidine and supplemented with 5 mM 3-AT.

## Supporting information

S1 Fig eGFP-DMP1 colocalizes with DMP1.2-mRFP in the PM and labels endosomes. (PDF)

S2 Fig Additional accumulation of DMP1.1-eGFP and DMP1.2-eGFP in the ER is age- and tissue-dependent. (PDF)

S3 Fig DMP1.1-eGFP and DMP1.2-eGFP targeting to the TP and the PM, respectively, is Golgi-dependent. (PDF)

S4 Fig Subcellular localization of DMP1-eGFP expressed from the native *Arabidopsis DMP1* promoter in tobacco leaves and *Arabidopsis* root tips. (PDF)

S5 Fig Investigation of DMP1 ER export using mutated versions of eGFP-DMP1. (PDF)

S6 Fig Interaction studies of DMP1 isoforms and determination of DMP1.1 and DMP1.2 orientation using rBiFc in transfected tobacco lower epidermis cells. (PDF)

S7 Fig Confirmation of DMP1 topology. (PDF)

S1 Table Primers, vectors, templates and cloning strategies. (PDF)
