# Correction: Dual-targeting of *Arabidopsis* DMP1 isoforms to the tonoplast and the plasma membrane

**DOI:** 10.1371/journal.pone.0178914

**Published:** 2017-06-02

**Authors:** The PLOS ONE Staff

Figs 1 and 7 appear incorrectly. Please view the correct Figs 1 and 7 here. The publisher apologizes for the errors.

**Fig 1:**
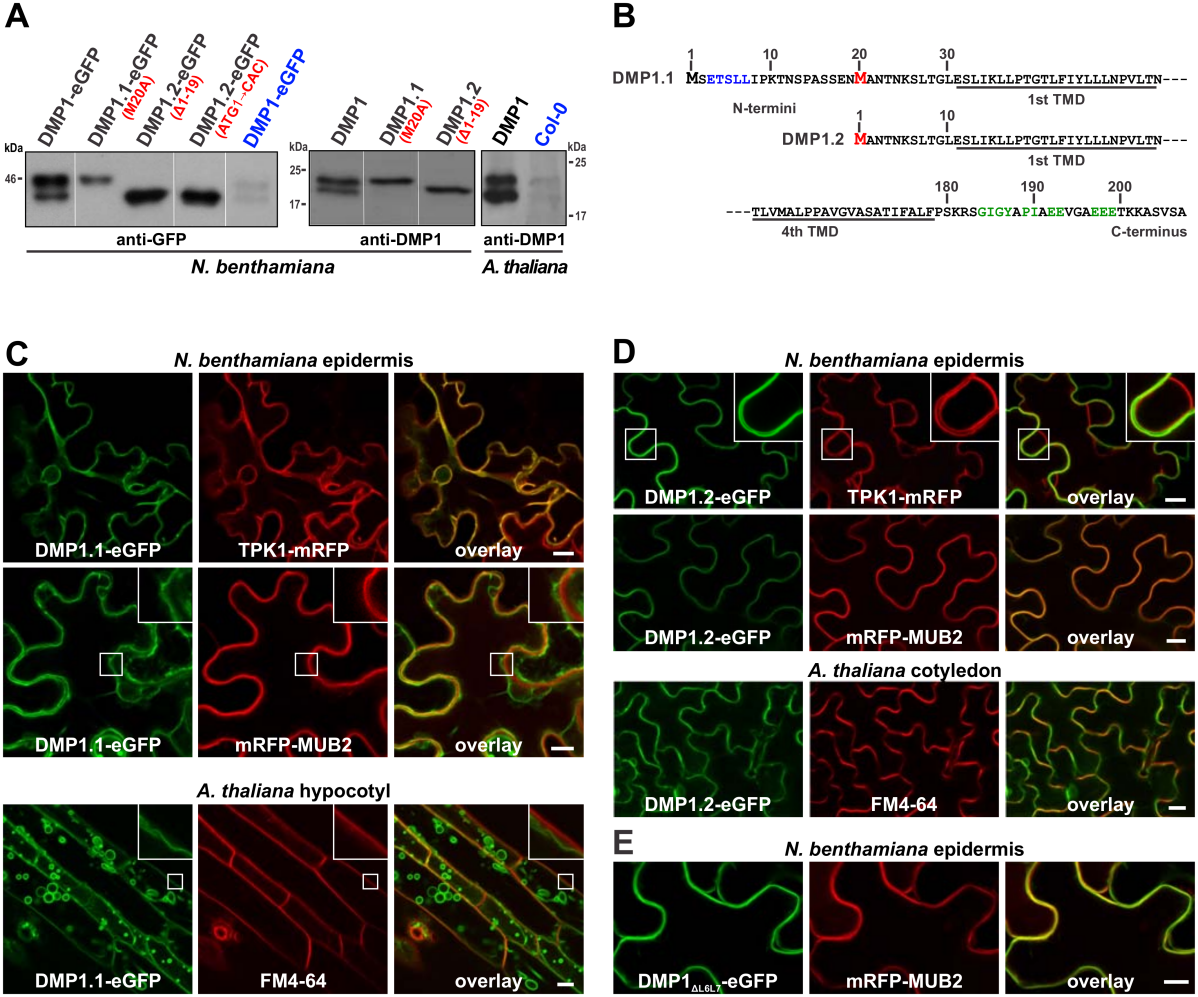
Identification and subcellular localization of DMP1 isoforms DMP1.1 and DMP1.2. (**A**) Western blot analysis of native and mutant DMP1 proteins transiently expressed in *Nicotiana benthamiana* abaxial leaf epidermis cells, in leaves of a transgenic *Arabidopsis thaliana* DMP1 overexpressor line, and in senescing leaves of wild-type *A*. *thaliana* Col-0 plants. Substitutions and deletions of the DMP1 open reading frame resulting in loss of the larger or the smaller isoform are indicated in red characters. Proteins were expressed from the 35S promoter (black characters) or the native *Arabidopsis DMP1* promoter (blue characters; ~20-times more protein was applied than in the other lanes). (**B**) Amino acid sequence of the DMP1.1 N-terminus with the second methionine in position 20 highlighted in red and a putative TP-targeting dileucine signal marked in blue letters (top line), the DMP1.2 N-terminus (center line), and the common C-terminus with putative ER-export signals highlighted in green letters (bottom line). TMD, transmembrane domain. (**C-E**) Determination by CLSM of (C) DMP1.1-eGFP, (D) DMP1.2-eGFP and (E) DMP1_ΔL6L7_-eGFP subcellular localization in coexpression experiments in transiently transfected tobacco abaxial epidermis cells (2 dpi) and transgenic *Arabidopsis* plants. The TP-located fusion protein TPK1-mRFP was used as TP marker and the PM-associated fusion protein mRFP-MUB2 as PM marker in tobacco. Staining of the PM in *Arabidopsis* plants was performed by incubating the cells for 10–15 min with the fluorescent dye FM4-64. Enlarged details in insets. Scale bars: 10 μm.

**Fig 7:**
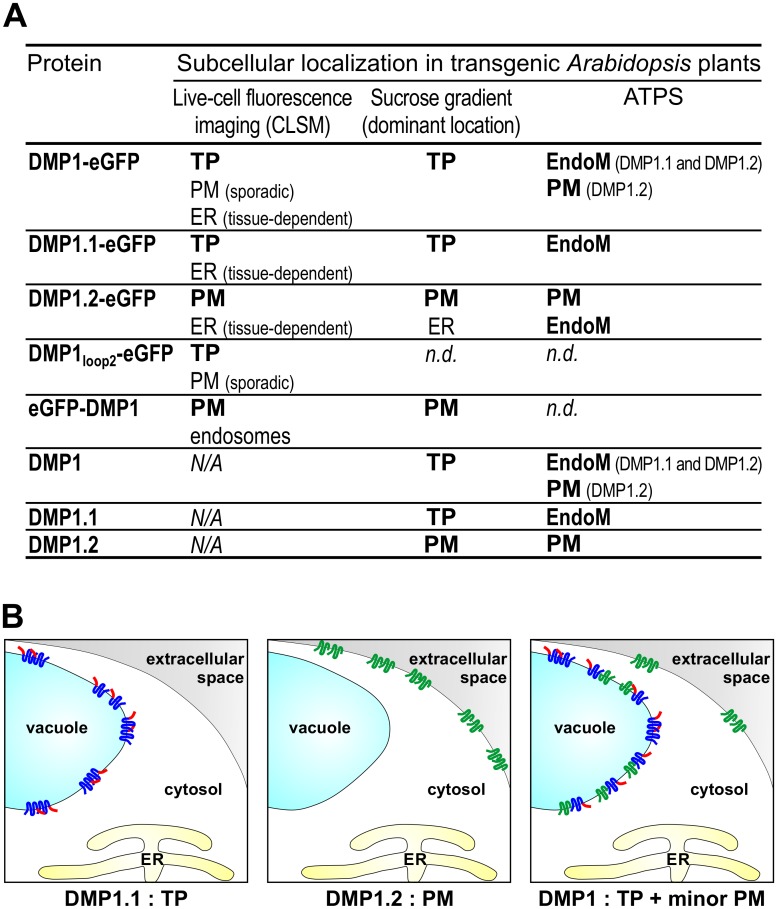
Overview and model. (**A**) Summary of subcellular location results. (**B**) Model for the localization of DMP1.1 and DMP1.2 proteins when individually or co-expressed. Individually expressed DMP1.1 is targeted to the TP and DMP1.2 to the PM. DMP1.2 is efficiently rerouted to the TP upon protein-protein interaction with DMP1.1 and only a minor fraction is targeted to the PM. Additional marginal accumulation of DMP1.1-eGFP and DMP1.2-eGFP in the ER membrane observed in some tissues is artifactual and not depicted. As both DMP1.1 and DMP1.2 trafficking to the TP or the PM is Brefeldin A-sensitive, DMP1.1-DMP1.2 protein-protein interaction presumably takes place in the ER membrane, Golgi and/or prevacuolar compartments before transit to either the TP or the PM.
