# Supplementary material for: Dual-targeting of Arabidopsis DMP1 isoforms to the tonoplast and the plasma membrane
Source: PLoS One. 2017 Apr 6;12(4):e0174062. doi: 10.1371/journal.pone.0174062 (PMC5383025; doi:10.1371/journal.pone.0174062)
Supplement: S1 Fig — (PDF) [file pone.0174062.s001.pdf]

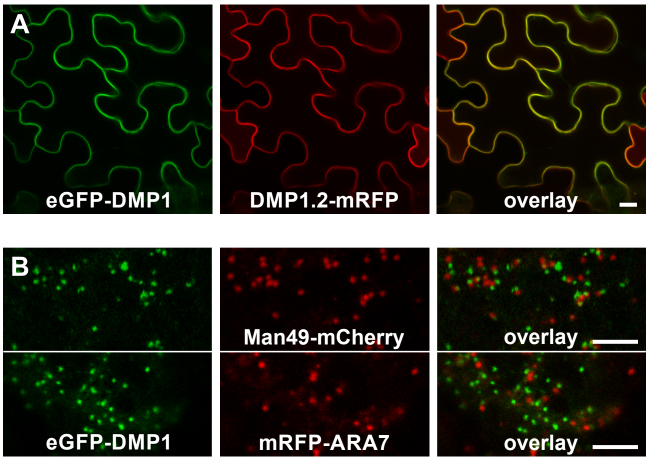

S1 Fig. eGFP-DMP1 colocalizes with DMP1.2-mRFP in the PM and labels endosomes. (**A**) The N-terminal fusion protein eGFP-DMP1 colocalizes with DMP1.2-mRFP in tobacco lower epidermis cells. (**B**) Additional Golgi-sized spherical structures were observed in tobacco at 2-3 dpi. These structures do neither correspond to Golgi vesicles labeled with Man49-mCherry nor to prevacuolar compartments (PVC) labeled with mRFP-ARA7 and might therefore correspond to vesicles of the transgolgi network (TGN). Scale bar: 10 µm
