# Supplementary material for: Dual-targeting of Arabidopsis DMP1 isoforms to the tonoplast and the plasma membrane
Source: PLoS One. 2017 Apr 6;12(4):e0174062. doi: 10.1371/journal.pone.0174062 (PMC5383025; doi:10.1371/journal.pone.0174062)
Supplement: S2 Fig — (PDF) [file pone.0174062.s002.pdf]

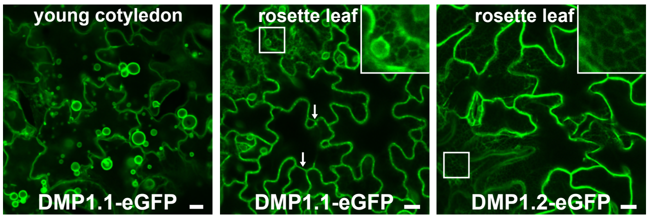

S2 Fig. Additional accumulation of DMP1.1-eGFP and DMP1.2-eGFP in the ER is age- and tissue-dependent. As described previously for DMP1-eGFP (Kasaras et al., 2012), DMP1.1-eGFP also accumulates in the tonoplast, tonoplastic bulbs and transvacuolar strands in young cotyledons. The additional accumulation in the ER is only weakly visible in young cotyledons, but intense in older tissues such as adult rosette leaves (enlarged in insets). Comparable changes are observed for DMP1.2-eGFP. The ER is only weakly visible in young cotyledons beside the PM (see Fig 1) but clearly visible in adult leaves. Scale bar: 10 µm.
