# Supplementary material for: Dual-targeting of Arabidopsis DMP1 isoforms to the tonoplast and the plasma membrane
Source: PLoS One. 2017 Apr 6;12(4):e0174062. doi: 10.1371/journal.pone.0174062 (PMC5383025; doi:10.1371/journal.pone.0174062)
Supplement: S3 Fig — (PDF) [file pone.0174062.s003.pdf]

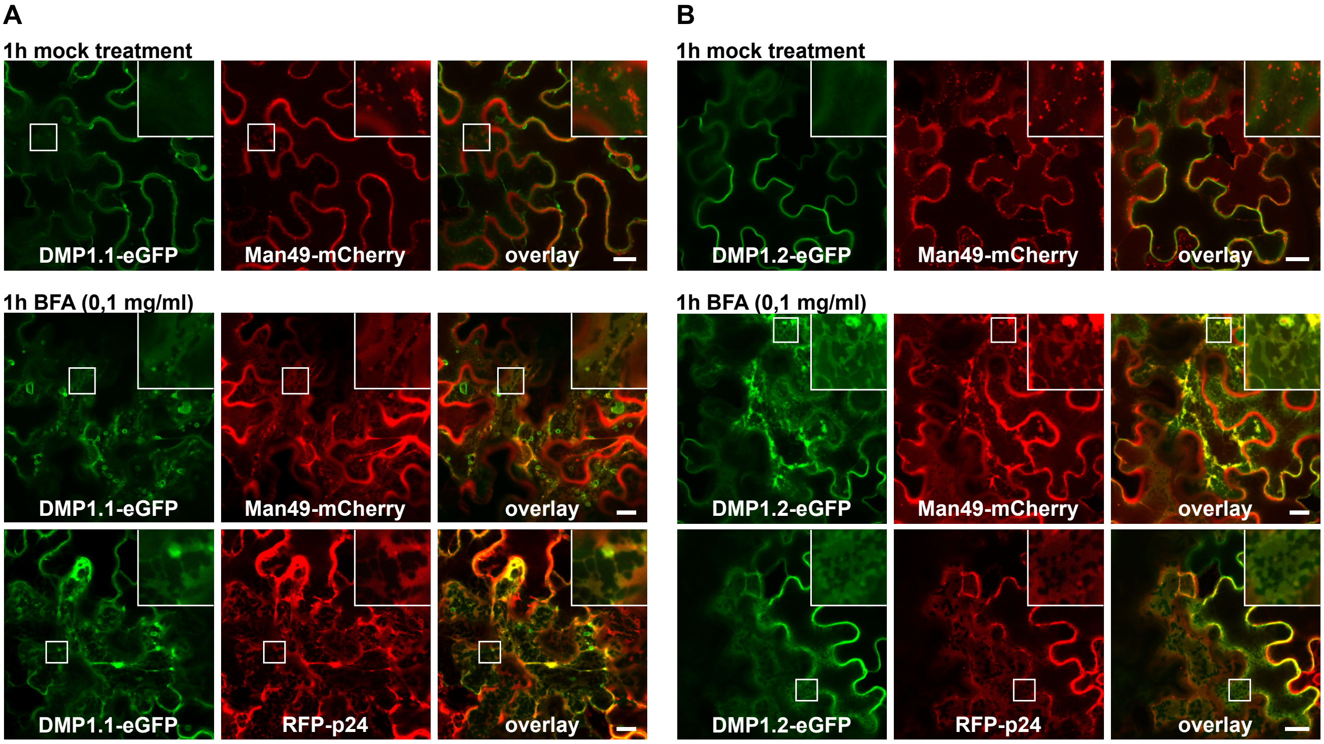

S3 Fig. DMP1.1-eGFP and DMP1.2-eGFP targeting to the TP and the PM, respectively, is Golgi-dependent. Tobacco abaxial epidermis cells co-expressing (**A**) DMP1.1-eGFP or (**B**) DMP1.2-eGFP with the Golgi marker Man49-mCherry were subjected to mock or Brefeldin A (BFA) treatment (0.1 mg/ml) for 1 h. As DMP1-eGFP accumulation in the ER membrane is weak at 2 dpi in tobacco epidermis cells [3], BFA and mock treatments were carried out at this time point. Besides their localization in the TP (DMP1.1-eGFP) or PM (DMP1.2-eGFP), both fusion proteins were not or only weakly found in the ER membrane upon mock treatment but accumulated in the ER upon BFA treatment. The accumulation of both fusion proteins in the ER membrane was confirmed in co-localization experiments with the ER marker RFP-p24. Scale bars: 10 µm.
