# Supplementary material for: Dual-targeting of Arabidopsis DMP1 isoforms to the tonoplast and the plasma membrane
Source: PLoS One. 2017 Apr 6;12(4):e0174062. doi: 10.1371/journal.pone.0174062 (PMC5383025; doi:10.1371/journal.pone.0174062)
Supplement: S4 Fig — (PDF) [file pone.0174062.s004.pdf]

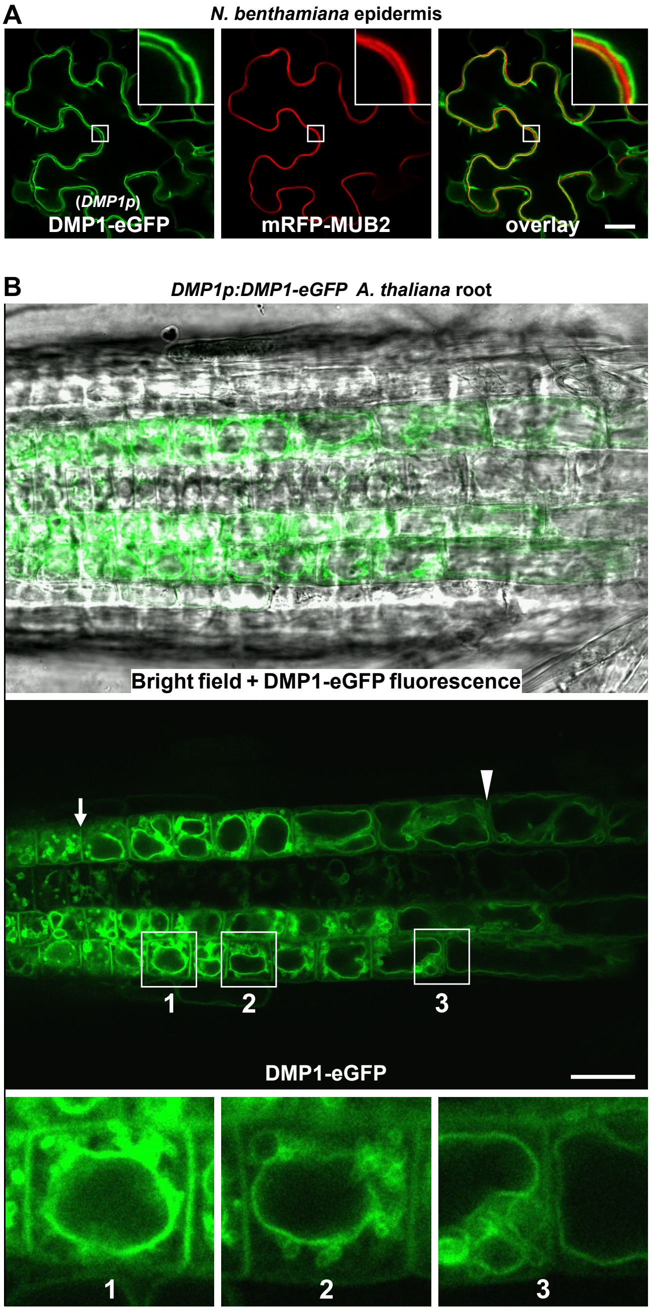


**S4 Fig. Subcellular localization of DMP1-eGFP expressed from the native *Arabidopsis DMP1* promoter in tobacco leaves and *Arabidopsis* root tips.** (**A**) DMP1-eGFP and mRFP-MUB2 fluorescence signals show clear separation in transiently transformed tobacco epidermis cells using CLSM, indicating no detectable labeling of the PM by DMP1.2-eGFP. (**B**) DMP1-eGFP fluorescence signals originate from vacuolar structures in the transition zone of primary roots in stably transformed *Arabidopsis* plants expressing DMP1-eGFP by the native *Arabidopsis DMP1* promoter. In meristematic, non-elongated cells, additional signals in the PM are detected whereas in elongating cells, signals originating from the PM are weak or not detectable (compare insets 1 to 3 and arrow with arrowhead). Scale bars: 20 µm.
