# Supplementary material for: Dual-targeting of Arabidopsis DMP1 isoforms to the tonoplast and the plasma membrane
Source: PLoS One. 2017 Apr 6;12(4):e0174062. doi: 10.1371/journal.pone.0174062 (PMC5383025; doi:10.1371/journal.pone.0174062)
Supplement: S5 Fig — (PDF) [file pone.0174062.s005.pdf]

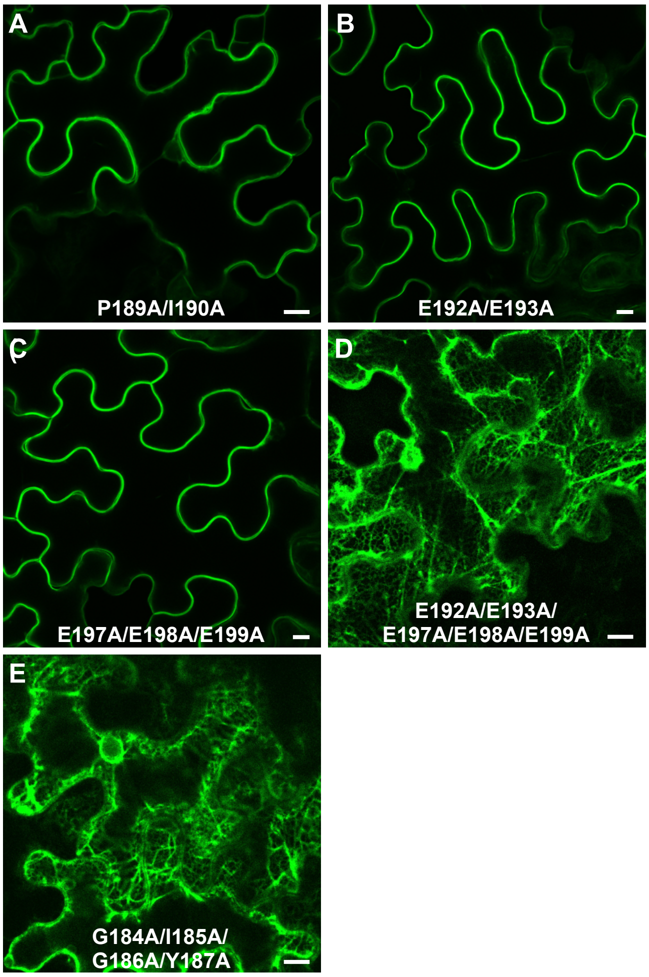

S5 Fig. Investigation of DMP1 ER export using mutated versions of eGFP-DMP1. Substitution of (**A**) the PI motif in position 189/190, (**B**) the aspartate residues in position 192/193 or (**C**) in position 197/198/199 by alanine residues does not affect traffic of eGFP-DMP1 to the PM. (**D**) Simultaneous mutation of all five aspartate residues leads to retention of eGFP-DMP1 in the ER membrane. (**E**) Mutation of the conserved motif GIGY to AAAA also impairs ER export of eGFP-DMP1. Scale bars: 10 µm.
