# Supplementary material for: Dual-targeting of Arabidopsis DMP1 isoforms to the tonoplast and the plasma membrane
Source: PLoS One. 2017 Apr 6;12(4):e0174062. doi: 10.1371/journal.pone.0174062 (PMC5383025; doi:10.1371/journal.pone.0174062)
Supplement: S6 Fig — (PDF) [file pone.0174062.s006.pdf]

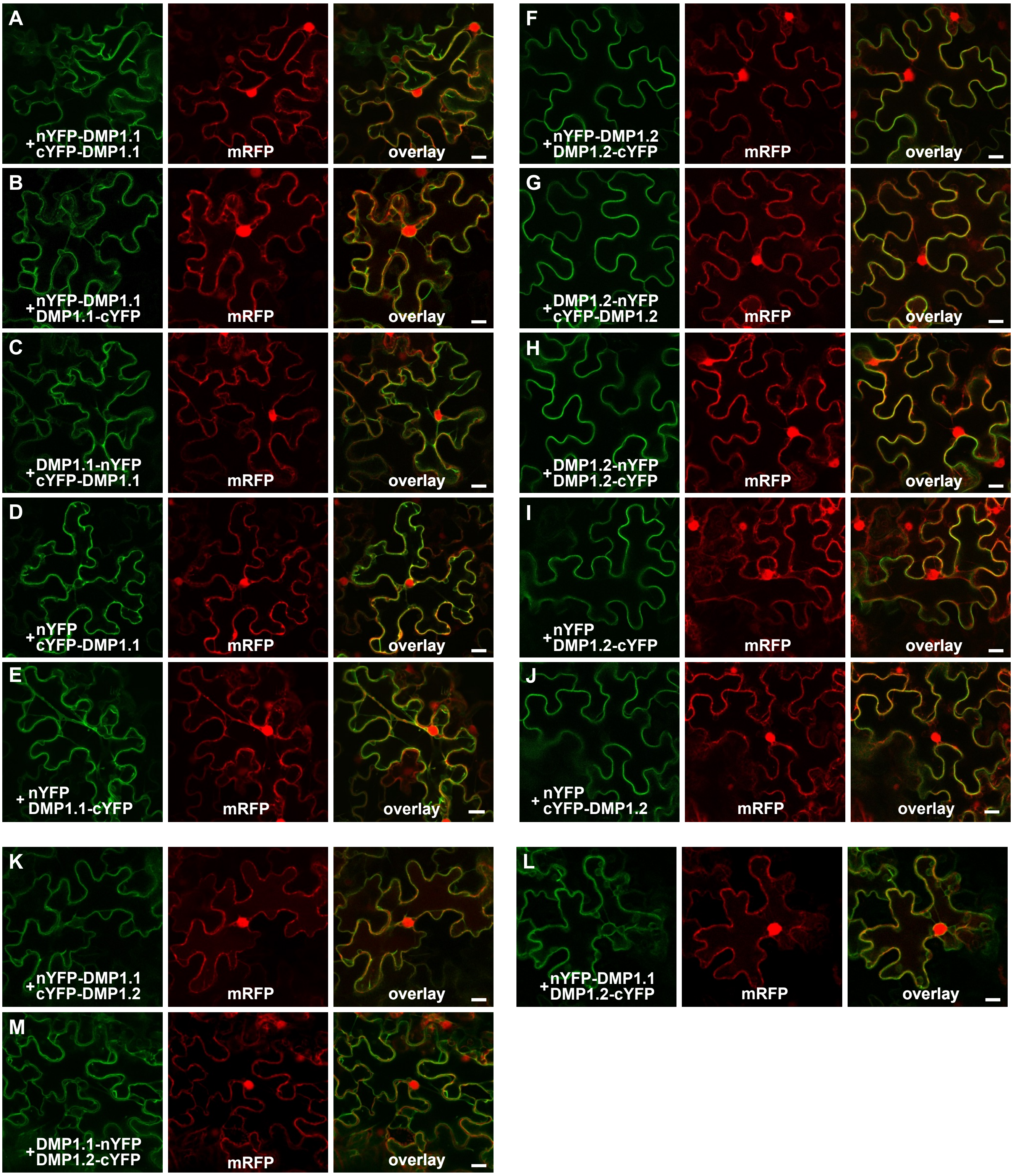

S6 Fig. Interaction studies of DMP1 isoforms and determination of DMP1.1 and DMP1.2 orientation using rBiFc in transfected tobacco lower epidermis cells. Left panels: Fluorescence signals of the indicated coexpressed fusion proteins containing the two YFP moieties (nYFP and cYFP) at 2 dpi in tobacco epidermis cells. Center panels: mRFP fluorescence signals visualizing the cytoplasm and the lumen of the nucleus. Right panels: superimposed YFP and mRFP signals. The three proteins for each assay were encoded on the same vector to ensure synchronized expression and equimolar protein levels. (**A**) nYFP-DMP1.1 + cYFP-DMP1.1, (**B**) nYFP-DMP1.1 + DMP1.1-cYFP, (**C**) DMP1.1-nYFP + cYFP-DMP1.1, (**D**) nYFP + cYFP-DMP1.1 and (**E**) nYFP + DMP1.1-cYFP show similar TP fluorescence patterns with apparent "wrapping" of the nucleus and larger organelles and the presence of transvacuolar strands. This indicates that all fusion proteins localize to the TP and that no detectable free cYFP or nYFP is released by proteolytic cleavage of fusion protein as no YFP fluorescence within the nucleus, colocalizing with free mRFP was observed. (**F**) nYFP-DMP1.2 + DMP1.2-cYFP, (**G**) DMP1.2-nYFP + cYFP-DMP1.2, (**H**) DMP1.2-nYFP + DMP1.2-cYFP, (**I**) nYFP + DMP1.2-cYFP and (**J**) nYFP + cYFP-DMP1.2 show similar PM fluorescence patterns with no "wrapping" of the nucleus or larger organelles, no tonoplastic "bulbs" and no transvacuolar strands. (**K**-**M**) The fluorescence signals of the three DMP1.1-DMP1.2 combinations can be clearly attributed to the TP, indicating rerouting of DMP1.2 to the TP upon interaction with DMP1.1. Scale bars: 10 µm
