# Supplementary material for: Dual-targeting of Arabidopsis DMP1 isoforms to the tonoplast and the plasma membrane
Source: PLoS One. 2017 Apr 6;12(4):e0174062. doi: 10.1371/journal.pone.0174062 (PMC5383025; doi:10.1371/journal.pone.0174062)
Supplement: S7 Fig — (PDF) [file pone.0174062.s007.pdf]

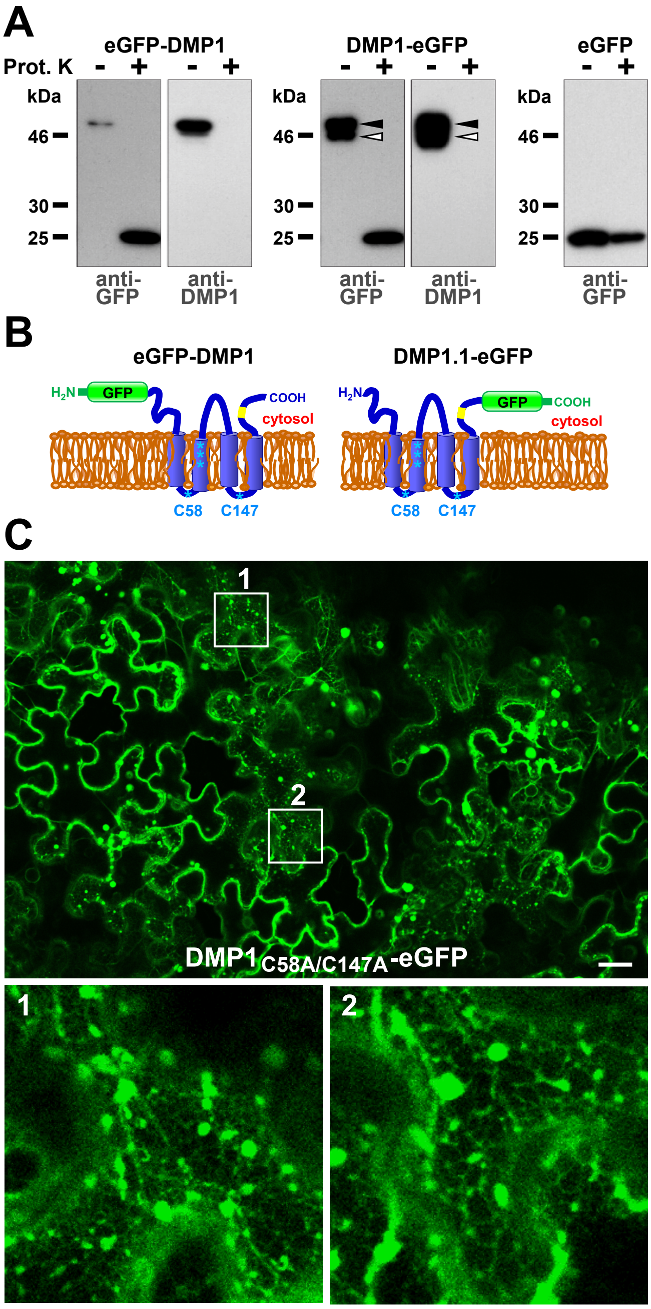

S7 Fig. Confirmation of DMP1 topology. (**A**) Untreated and Proteinase K-treated microsomes from tobacco leaves transiently expressing DMP1-eGFP, eGFP-DMP1 or eGFP were analysed by Western blotting using anti-GFP and anti-DMP1 antibodies, the latter being directed against a DMP1 C-terminal peptide (**B**, yellow segment). Patterns obtained before and after proteinase K treatment for both eGFP-DMP1 and DMP1-eGFP proteins suggest that eGFP faces the cytoplasm. From both fusion proteins a Proteinase K digestion product of the same size as free eGFP (26.9 kDa, right panel) was detected with anti-eGFP antibody, suggesting the release of free eGFP. For both fusion proteins, no DMP1 moiety was detected using anti-DMP1 antibody, indicating exposure of the DMP1 epitope at the cytosolic surface and thus digestion by the protease. If the DMP1 C-terminus were facing the lumen of the microsomes, DMP1-eGFP would be expected to be cleaved into a 32.2 kDa product consisting of eGFP, the C-terminus of DMP1 and its last TMD. The respective eGFP-DMP1 digestion product should consist of eGFP, the N-terminus of DMP1 and the first TMD with a size of 32.6 kDa. The lack of these products demonstrates that both N- and C-termini are facing the cytosol as depicted in (**B**). The five cysteine residues in position 58, 73, 77, 81 and 147 of DMP1 primary sequence are depicted by pale blue asterisks. (**C**) Substitution of C58 and C147 by alanine residues, yielding DMP1$_{C58A/C147A}$-eGFP, impairs proper trafficking to the TP as the mutated fusion protein labels the ER network and aggregates of various sizes apparently associated with the ER (see insets 1 and 2). Scale bar: 20 µm
