# Supplementary material for: Dual-targeting of Arabidopsis DMP1 isoforms to the tonoplast and the plasma membrane
Source: PLoS One. 2017 Apr 6;12(4):e0174062. doi: 10.1371/journal.pone.0174062 (PMC5383025; doi:10.1371/journal.pone.0174062)
Supplement: S1 Table — (PDF) [file pone.0174062.s008.pdf]

# Table S1. Plasmid constructs.

| Generation of constructs by conventional cloning using restriction enzymes and T4 DNA ligase | | | | | | |
|---|---|---|---|---|---|---|
| **Protein** | **Construct** | **Forward Primer (FP)** | **Reverse Primer (RP)** | **Template** | **Vector** | **Remarks** |
| **DMP1.1-eGFP (DMP1$_{M20A}$-eGFP)** | *35S:DMP1.1-eGFP* | FP1: CGGTCTAGAATGTCCGAAACTTCTTTGCTC<br>FP2: CCAGCTTCATCAGAAAACgccgcaaacacaaacaaatccctc | RP1: gagggatttgtttgtgtttgcggcGTTTTCTGATGAAGCTGG<br>RP2: AACTGCAGCGGCAGAGACCGAGGCTTTC | *35S:DMP1-eGFP* | *pPGTkan3* | First independent PCR reactions with FP1/RP1 and FP2/RP2 and *35S:DMP1-eGFP* as template. Second PCR using FP1/RP2 and previous overlapping PCR amplificates as template. Digestion of final PCR product and vector with XbaI and PstI followed by ligation |
| **DMP1.2-eGFP (DMP1$_{\Delta 1\text{-}19}$-eGFP)** | *35S:DMP1.2-eGFP* | CGGTCTAGAATGGCAAACACAAACAAATCC | AACTGCAGCGGCAGAGACCGAGGCTTTC | *35S:DMP1-eGFP* | *pPGTkan3* | Digestion of PCR product and vector with XbaI and PstI followed by ligation |
| **DMP1.2-eGFP (ATG1→CAC)** | *35S:DMP1.2-eGFP* | ACGGTCTAGACACTCCGAAACTTCTTTGCTC | AACTGCAGCGGCAGAGACCGAGGCTTTC | | | |
| **DMP1$_{\Delta L6L7}$-eGFP** | *35S:DMP1$_{\Delta L6L7}$-eGFP* | ACGGTCTAGAATGTCCGAAACTTCTATACCCAAAACAAACTCT | AACTGCAGCGGCAGAGACCGAGGCTTTC | | | |
| **eGFP-DMP1** | *35S:eGFP-DMP1* | CGGTCTAGAATGTCCGAAACTTCTTTGCTC | AACTGCAGTTAGGCAGAGACCGAGGCTTTC | *35S:DMP1-eGFP* | *pNGTkan3* | Digestion of PCR product and with XbaI and PstI followed by ligation |
| **DMP1$_{loop2}$-eGFP** | *35S:DMP1$_{loop2}$-eGFP* | CGGTCTAGAATGTCCGAAACTTCTTTGCTC | AACTGCAGTTAGGCAGAGACCGAGGCTTTC | pPGTkan3 and *35S:DMP1-eGFP* | *pPTkan3* | See additional remarks |
| **Cub-DMP1.1** | *CYC1:LexA-VP16-Cub-DMP1.1* | TCAACTGCAGATGTCCGAAACTTCTTTGCTC | GAGACCATGGTTAGGCAGAGACCGAGGCTTTC | *35S:DMP1-eGFP* | *pBT3-N* | Digestion of PCR product and vector with PstI and NcoI followed by ligation |
| **DMP1.2-mRFP** | *35S:DMP1.2-mRFP* | ACGGTCTAGAATGGCAAACACAAACAAATCC | TAGACTCGAGGGCAGAGACCGAGGCTTTCTT | *35S:DMP1-eGFP* | *pPRTkan* | Digestion of PCR product and vector with XbaI and XhoI followed by ligation |
| **eGFP-DMP1 (G184A/I185A/G186A /Y187A)** | *35S:eGFP-DMP1$_{G184A/I185A/G186A/Y187A}$* | FP1: see *35S:eGFP-DMP1* FP<br>FP2: CCGAGCAAACGAAGTGCAGCAGCAGCAGCGCCAATTGCT | RP1: AGCAATTGGCGCTGCTGCTGCTGCACTTCGTTTGCTCGG<br>RP2: see *35S:eGFP-DMP1* RP | *35S:eGFP-DMP1* | *pNGTkan3* | First independent PCR reactions with FP1/RP1 and FP2/RP2 and *35S:eGFP-DMP1* as template. Second PCR using FP1/RP2 and previous overlapping PCR amplificates as template. Digestion of final PCR product and vector with XbaI and PstI followed by ligation |
| **eGFP-DMP1 (P189A/I190A)** | *35S:eGFP-DMP1$_{P189A/I190A}$* | FP1: see *35S:eGFP-DMP1* FP<br>FP2: CGAAGTGGAATCGGGTACGCGGCAGCAGCTGAGGAGGTAGGTGCTGAA | RP1: TTCAGCACCTACCTCCTCAGCTGCTGCCGCGTACCCGATTCCACTTCG<br>RP2: see *35S:eGFP-DMP1* RP | *35S:eGFP-DMP1* | *pNGTkan3* | Comparable two-step strategy as above. Digestion of final PCR product and vector with XbaI and PstI followed by ligation |
| **eGFP-DMP1 (E192A/E193A)** | *35S:eGFP-DMP1$_{E192A/E193A}$* | FP1: see *35S:eGFP-DMP1* FP<br>FP2: GGGTACGCGCCAATTGCTGCGGCGGTAGGTGCTGAAGAGGAG | RP1: CGCCGCAGCAATTGGCGCGTACCC<br>RP2: see *35S:eGFP-DMP1* RP | | | |
| **eGFP-DMP1 (E197A/E198A/E199A)** | *35S:eGFP-DMP1$_{E197A/E198A/E199A}$* | FP1: see *35S:eGFP-DMP1* FP<br>FP2: GCTGAGGAGGTAGGTGCTGCGGCGGCGACCAAGAAAGCCTCGGTC | RP1: CGCCGCCGCAGCACCTACCTCCTCAGC<br>RP2: see *35S:eGFP-DMP1* RP | | | |
| **eGFP-DMP1 (E192A/E193A/E197A/ E198A/E199A)** | *35S:eGFP-DMP1 $_{E192A/E193A/E197A/E198A/E199A}$* | CGGTCTAGAATGTCCGAAACTTCTTTGCTC | TTCTGCAGCGGCAGAGACCGAGGCtttcttggtggcggcggcagcacctacggcggcAGCAATTGGCGCGTACCCGATTCC | | | |
| **DMP1-eGFP (C58A/C147A)** | *35S:DMP1$_{C58A/C147A}$-eGFP* | FP1: see *35S:DMP1.1-eGFP* FP<br>FP2: ACTAACGACGGTGAAGCATCCACAGGTAATAAG<br>FP3: GCTAATACCGCGAGCGCATTTTATCCTCGGTTC | RP1: CTTATTACCTGTGGATGCTTCACCGTCGTTAGT<br>RP2: GAACCGAGGATAAAATGCGCTCGCGGTATTAGC<br>RP3: see *35S:DMP1.1-eGFP* RP | *35S:DMP1-eGFP* | *pPGTkan3* | First independent PCR reactions with FP1/RP1, FP2/RP2 and FP3/RP3 and *35S:DMP1-eGFP* as template. Second PCR using FP1/RP3 and previous overlapping PCR amplificates as template. Digestion of final PCR product and vector with XbaI and PstI followed by ligation |

**Additional remarks:**
**Generation of *35S:DMP1$_{loop2}$-eGFP***. Three PCR fragments were amplified in parallel. eGFP was amplified with GAGACTCGAGGGAGGTGGAGGTATGGTGAGCAAGGGCGAGGAG and GAGACCATGGTGCACCTCCTGCCTTGTACAGCTCGTCCATGCC using pPGTkan3 as template to generate XhoI-4xGly-eGFP(w/o Stop)-Ala-Gly-Glu-Ala-NcoI. DMP1 was amplified as two fragments with two primer pairs and *35S:DMP1-eGFP* as template: the first one consisting of XbaI-DMP1(1-108)-XhoI using FP listed above and GAGACTCGAGctccgcgtaagtccaaagacc and the second one consisting of NcoI-DMP1(109-207)-PstI using GAGACCATGGccaggatccgtggatttatcc and LP listed above. DMP1(1-108)-eGFP-DMP1(109-207) was generated by XhoI-NcoI digestion followed by ligation. This construct was subsequently cloned into pPGTkan3 by XbaI-PstI restriction followed by ligation giving rise to *35S:DMP1$_{loop2}$-eGFP*.

## Generation of entry clones by BP reactions (Gateway technology)

| Entry Clone | Forward Primer (FP) | Reverse Primer (RP) | Template | Shuttle Vector |
|---|---|---|---|---|
| ***DMP1-pDONR222*** | GGGGACAAGTTTGTACAAAAAAGCAGGCTTGATGTCCGAAACTTCTTTGCTC | GGGGACCACTTTGTACAAGAAAGCTGGGTTGGCAGAGACCGAGGCTTTC | *35S:DMP1-eGFP* | *pDONR222* |
| ***DMP1.1-pDONR222*** | See *35S:DMP1* | See *35S:DMP1* | *35S:DMP1.1-eGFP* | *pDONR222* |
| ***DMP1.2-pDONR222*** | GGGGACAAGTTTGTACAAAAAAGCAGGCTTGATGGCAAACACAAACAAATCC | GGGGACCACTTTGTACAAGAAAGCTGGGTACYAGGCAGAGACCGAGGCTTTCTT | *35S:DMP1-eGFP* | *pDONR222* |
| ***DMP2-pDONR222*** | GGGGACAAGTTTGTACAAAAAAGCAGGCTTGATGTCGAAAACATTCAAAGCC | GGGGACCACTTTGTACAAGAAAGCTGGGTACYATTTCATCTCGGAAGCATC | gDNA | *pDONR222* |
| ***DMP7-pDONR222*** | GGGGACAAGTTTGTACAAAAAAGCAGGCTTGATGGAGGAGACGAAGCAGTCA | GGGGACCACTTTGTACAAGAAAGCTGGGTACYATTCTTTGGTAAGGGGAGA | cDNA | *pDONR222* |
| ***DMP1.1(Stop)-pDONR221-L1L4*** | GGGGACAAGTTTGTACAAAAAAGCAGGCTTAATGTCCGAAACTTCTTTGCTC | GGGGACAACTTTGTATAGAAAAGTTGGGTGTTAGGCAGAGACCGAGGCTTT | *DMP1.1-pDONR222* | *pDONR221-P1P4* |
| ***DMP1.1(w/o Stop)-pDONR221-L1L4*** | Same as FP *DMP1.1(Stop)-pDONR221-L1L4* | GGGGACAACTTTGTATAGAAAAGTTGGGTGGGCAGAGACCGAGGCTTTCTT | *DMP1.1-pDONR222* | *pDONR221-P1P4* |
| ***DMP1.2(Stop)-pDONR221-L1L4*** | GGGGACAAGTTTGTACAAAAAAGCAGGCTTAATGGCAAACACAAACAAATCC | Same as RP *DMP1.1(Stop)-pDONR221-L1L4* | *DMP1.2-pDONR222* | *pDONR221-P1P4* |
| ***DMP1.2(w/o Stop)-pDONR221-L1L4*** | Same as FP *DMP1.2(Stop)-pDONR221-L1L4* | Same as RP *DMP1.1(w/o Stop)-pDONR221-L1L4* | *DMP1.2-pDONR222* | *pDONR221-P1P4* |
| ***DMP1.1(Stop)-pDONR221-L3L2*** | GGGGACAACTTTGTATAATAAAGTTGTAATGTCCGAAACTTCTTTGCTC | GGGGACCACTTTGTACAAGAAAGCTGGGTTTTAGGCAGAGACCGAGGCTTT | *DMP1.1-pDONR222* | *pDONR221-P3P2* |
| ***DMP1.1(w/o Stop)-pDONR221-L3L2*** | Same as FP *DMP1.1(Stop)-pDONR221-L3L2* | GGGGACCACTTTGTACAAGAAAGCTGGGTTGGCAGAGACCGAGGCTTTCTT | *DMP1.1-pDONR222* | *pDONR221-P3P2* |
| ***DMP1.2(Stop)-pDONR221-L3L2*** | GGGGACAACTTTGTATAATAAAGTTGTAATGGCAAACACAAACAAATCC | Same as RP *DMP1.1(Stop)-pDONR221-L3L2* | *DMP1.2-pDONR222* | *pDONR221-P3P2* |
| ***DMP1.2(w/o Stop)-pDONR221-L3L2*** | Same as FP *DMP1.2(Stop)-pDONR221-L3L2* | Same as RP *DMP1.1(w/o Stop)-pDONR221-L3L2* | *DMP1.2-pDONR222* | *pDONR221-P3P2* |

## Generation of expression vectors by LR reactions between entry clones and destination vectors (Gateway technology)

| Protein(s) | Construct | Entry Clone 1 | Entry Clone 2 | Destination vector |
|---|---|---|---|---|
| **DMP1** | *35S:DMP1* | *DMP1-pDONR222* | - | *pB2GW7* |
| **DMP1.1** | *35S:DMP1.1* | *DMP1.1-pDONR222* | - | *pB2GW7* |
| **DMP1.2** | *35S:DMP1.2* | *DMP1.2-pDONR222* | - | *pB2GW7* |
| **NubG-DMP1.1** | *ADH1:NubG-DMP1.1-3xHA* | *DMP1.1-pDONR222* | - | *NX32_GW* |
| **NubG-DMP1.2** | *ADH1:NubG-DMP1.2-3xHA* | *DMP1.2-pDONR222* | - | *NX32_GW* |
| **NubG-DMP2** | *ADH1:NubG-DMP2-3xHA* | *DMP2-pDONR222* | - | *NX32_GW* |
| **NubG-DMP7** | *ADH1:NubG-DMP7-3xHA* | *DMP7-pDONR222* | - | *NX32_GW* |
| **NubI-DMP2** | *ADH1:NubI-DMP2-HA* | *DMP2-pDONR222* | - | *NWTX_GW* |
| **NubI-DMP7** | *ADH1:NubI-DMP7-HA* | *DMP7-pDONR222* | - | *NWTX_GW* |
| **nYFP-DMP1.1 + cYFP-DMP1.1** | *35Ω:nYFP-HA-DMP1.1/cYFP-Myc-DMP1.1/mRFP* | *DMP1.1(Stop)-pDONR221-L3L2* | *DMP1.1(Stop)-pDONR221-L1L4* | *pBiFCt-2in1-NN* |
| **nYFP-DMP1.1 + DMP1.1-cYFP** | *35Ω:nYFP-HA-DMP1.1/DMP1.1-Myc-cYFP/mRFP* | *DMP1.1(Stop)-pDONR221-L3L2* | *DMP1.1(w/o Stop)-pDONR221-L1L4* | *pBiFCt-2in1-NC* |
| **nYFP + cYFP-DMP1.1** | *35Ω:nYFP-HA/cYFP-Myc-DMP1.1/mRFP* | - | *DMP1.1(Stop)-pDONR221-L1L4* | *pBiFCt-2in1-NN* |
| **DMP1.1-nYFP + cYFP-DMP1.1** | *35Ω:DMP1.1-HA-nYFP/cYFP-Myc-DMP1.1/mRFP* | *DMP1.1(w/o Stop)-pDONR221-L3L2* | *DMP1.1(Stop)-pDONR221-L1L4* | *pBiFCt-2in1-CN* |

| | | | | |
|---|---|---|---|---|
| **DMP1.1-nYFP + DMP1.1-cYFP** | *35Ω:DMP1.1-HA-nYFP/DMP1.1-Myc-cYFP/mRFP* | *DMP1.1(w/o Stop)-pDONR221-L3L2* | *DMP1.1(w/o Stop)-pDONR221-L1L4* | *pBiFCt-2in1-CC* |
| **nYFP + DMP1.1-cYFP** | *35Ω:nYFP-HA/DMP1.1-Myc-cYFP/mRFP* | - | *DMP1.1(w/o Stop)-pDONR221-L1L4* | *pBiFCt-2in1-NC* |
| **nYFP-DMP1.2 + cYFP-DMP1.2** | *35Ω:nYFP-HA-DMP1.2/cYFP-Myc-DMP1.2/mRFP* | *DMP1.2(Stop)-pDONR221-L3L2* | *DMP1.2(Stop)-pDONR221-L1L4* | *pBiFCt-2in1-NN* |
| **nYFP-DMP1.2 + DMP1.2-cYFP** | *35Ω:nYFP-HA-DMP1.2/DMP1.2-Myc-cYFP/mRFP* | *DMP1.2(Stop)-pDONR221-L3L2* | *DMP1.2(w/o Stop)-pDONR221-L1L4* | *pBiFCt-2in1-NC* |
| **nYFP + cYFP-DMP1.2** | *35Ω:nYFP-HA/cYFP-Myc-DMP1.2/mRFP* | - | *DMP1.2(Stop)-pDONR221-L1L4* | *pBiFCt-2in1-NN* |
| **DMP1.2-nYFP + cYFP-DMP1.2** | *35Ω:DMP1.2-HA-nYFP/cYFP-Myc-DMP1.2/mRFP* | *DMP1.2(w/o Stop)-pDONR221-L3L2* | *DMP1.2(Stop)-pDONR221-L1L4* | *pBiFCt-2in1-CN* |
| **DMP1.2-nYFP + DMP1.2-cYFP** | *35Ω:DMP1.2-HA-nYFP/DMP1.2-Myc-cYFP/mRFP* | *DMP1.2(w/o Stop)-pDONR221-L3L2* | *DMP1.2(w/o Stop)-pDONR221-L1L4* | *pBiFCt-2in1-CC* |
| **nYFP + DMP1.2-cYFP** | *35Ω:nYFP-HA/DMP1.2-Myc-cYFP/mRFP* | - | *DMP1.2(w/o Stop)-pDONR221-L1L4* | *pBiFCt-2in1-NC* |
| **nYFP-DMP1.1 + cYFP-DMP1.2** | *35Ω:nYFP-HA-DMP1.1/cYFP-Myc-DMP1.2/mRFP* | *DMP1.1(Stop)-pDONR221-L3L2* | *DMP1.2(Stop)-pDONR221-L1L4* | *pBiFCt-2in1-NN* |
| **nYFP-DMP1.1 + DMP1.2-cYFP** | *35Ω:nYFP-HA-DMP1.1/DMP1.2-Myc-cYFP/mRFP* | *DMP1.1(Stop)-pDONR221-L3L2* | *DMP1.2(w/o Stop)-pDONR221-L1L4* | *pBiFCt-2in1-NC* |
| **DMP1.1-nYFP + cYFP-DMP1.2** | *35Ω:DMP1.1-HA-nYFP/cYFP-Myc-DMP1.2/mRFP* | *DMP1.1(w/o Stop)-pDONR221-L3L2* | *DMP1.2(Stop)-pDONR221-L1L4* | *pBiFCt-2in1-CN* |
| **DMP1.1-nYFP + DMP1.2-cYFP** | *35Ω:DMP1.1-HA-nYFP/DMP1.2-Myc-cYFP/mRFP* | *DMP1.1(w/o Stop)-pDONR221-L3L2* | *DMP1.2(w/o Stop)-pDONR221-L1L4* | *pBiFCt-2in1-CC* |
